# Synthesis and Evaluation of a Series of Ni(II) Complexed Nucleophilic Glycine Equivalents

**DOI:** 10.1021/acsomega.5c07357

**Published:** 2025-11-06

**Authors:** Audrey Jergensen, Emily Burgess, Mackenzie Bergagnini, Delaney McDonald, Shawna B. Ellis, Trevor K. Ellis

**Affiliations:** Department of Chemistry and Physics, 8452 Southwestern Oklahoma State University, 100 Campus Drive, Weatherford, Oklahoma 73096, United States

## Abstract

The synthesis of a series of Ni­(II) complexed glycine Schiff bases as well as the appropriate precursors is described. Included in this series is a new nucleophilic glycine equivalent, which includes an electron withdrawing chlorine atom which stabilizes the extended enolate necessary for alkylation of the glycine Schiff base. Additionally, the reactivity of this system was evaluated and compared to previous iterations of these complexes for the substitution of one or both protons of the glycine methylene moiety. The substitution of the first hydrogen was evaluated through a series of competitive benzylation reactions with benzyl bromide under phase transfer conditions with aqueous potassium hydroxide as the base. The substitution of both hydrogen atoms from the glycine moiety was evaluated by a similar process through bis-propargylation of the methylene group. It was found that this new series of complexes is similar in reactivity to the more synthetically complicated complexes, which incorporate a trifluoromethyl group for stabilization.

## Graphical abstract

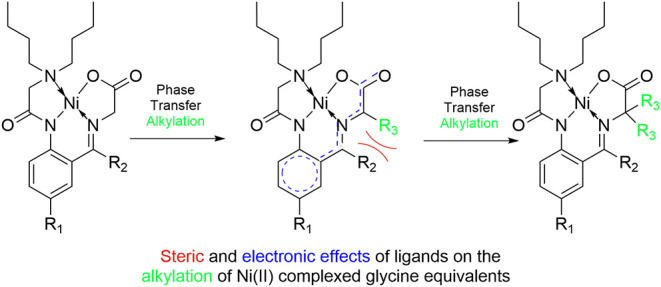

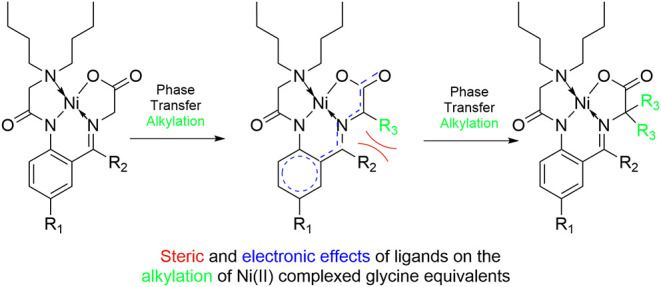

## Introduction

α-Amino acids are some of the most basic and universal building blocks in living organisms. These simple but powerful molecules are responsible for many essential biological functions. While much of the interest in α-amino acids focuses on those commonly found in proteins and enzymes, there are also a large number of noncanonical or unnatural amino acids that play vital roles in nonproteinogenic functions of living organisms, pharmaceutical therapies (both in their free form and as a component of larger systems), as well as a tool for investigation of protein form and function.^1–3^ Of particular interest to the pharmaceutical field is α,α-disubstituted amino acids due to the similarity of the quaternary center to many natural products.^4^ However, the steric hindrance around this center and the complexity of modifying the α-carbon without affecting the amine or carboxylic acid groups makes the synthesis of these molecules increasingly difficult and limited.^4^

To combat these issues, a variety of methods have been utilized. Methods currently used to synthesize α,α-disubstituted amino acids include total synthetic methods such as the Strecker Reaction, Bucherer–Bergs Reaction, CO_2_ Fixation, Visible-Light-Mediated Photocatalysis, as well as partial synthetic approaches such as the modification of Nucleophilic Glycine Equivalents and Enolate Alkylation.^5–10^ Each of these methods has its benefits and drawbacks. For Strecker synthesis, the method is applicable to a wide array of substrates and allows access to both mono- and disubstituted amino acids, but it often has low yields, producing racemic mixtures, and the application of toxic agents, such as cyanide, which are required for the reaction.^6^ The Bucherer–Bergs reaction allows for a wide array of starting materials and has biological relevance, but it is slow and low yielding. Additionally like the Strecker reaction, it requires the use of toxic reagents.^7^ CO_2_ Fixation is a green chemistry reaction and uses mild conditions, but it has a limited substrate scope and low scalability and requires specialized equipment and catalysts.^8^ Visible-light-mediated photocatalysis applies mild conditions, a broad substrate tolerance, and stereoselectivity, but it has limited scalability and requires careful control and the use of photocatalysts.^9^

However, to date, the modification of the glycine methylene unit of nucleophilic glycine equivalents remains among the most widely applicable and efficient methods for the synthesis of α-amino acid derivatives.^10^ Among these nucleophilic glycine equivalents, the Ni­(II) coordinated Schiff bases of glycine have proven useful for the preparation of a wide array of these versatile materials.^11^ Several generations of these modular metal complexes have been introduced, each designed to address the demands of specific methods of homologation. These design alterations have produced complexes that allow for the preparation of symmetrical but sterically constrained α-amino acids through bis-alkylation of the methylene unit, asymmetric preparation of β-substituted pyroglutamic acids through topographically controlled Michael Addition Reactions, asymmetrically selective monoalkylated α-amino acids by application of chiral ligands, as well as the more recent developments of these complexes for the Dynamic Kinetic Resolution of racemic α-amino acids (Figure 1).^12–14^ However, despite all of the modifications that have been made for these complexes, very few have been directed toward altering the p*K*_a_ of the methylene unit of the nucleophilic glycine equivalent.^15^ Therefore, in order to address this issue, we have undertaken studies to design, synthesize, and analyze the reactivity of new derivatives of these Ni­(II) complexes with increased acidity of the methylene protons of the glycine subunit.

**Figure 1:**
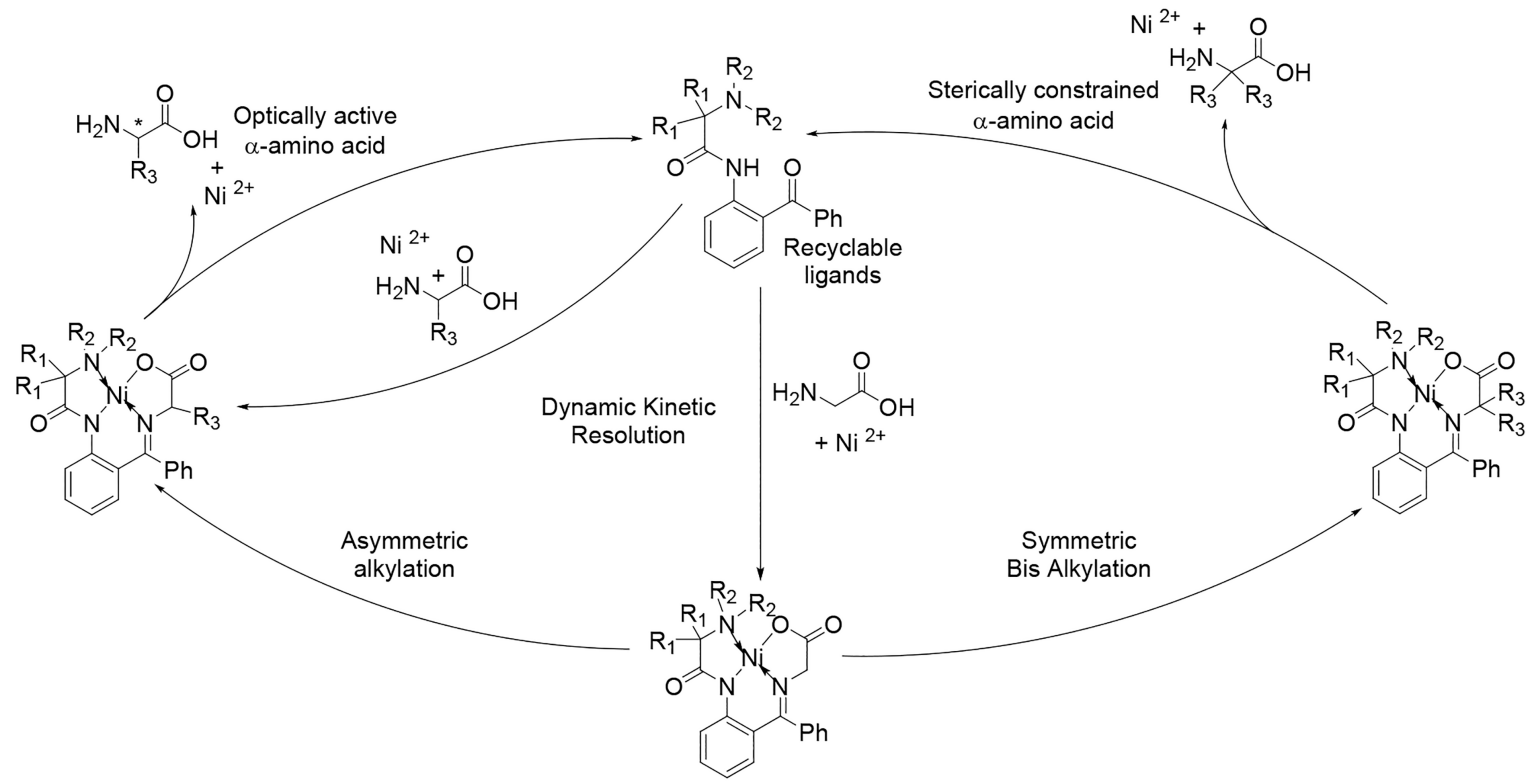
Applications of modular Ni­(II) complexes of α-amino acid Schiff bases.

Evaluating the skeleton of the glycine equivalent and the way the anion that results from deprotonation of the methylene unit of the glycine is stabilized through resonance, it was apparent that the p*K*_a_ may be perturbed by the incorporation of electron withdrawing moieties on the aromatic rings of the benzophenone system (Figure 2).^16^ Therefore, to investigate this theory, commercially available 2-aminobenzophenone and 2-amino-5-chlorobenzophenone as well as the more intriguing but more difficult to obtain 2-[4-(trifluoromethyl)­benzoyl]­analine was selected as benzophenone modules for the study. The next goal was to increase the reactivity with respect to homologation of the methylene moiety of the glycine Schiff base. It was envisioned that the chlorine and, to a greater extent, the trifluoromethyl groups would impart a large stabilizing effect on the resulting extended resonance of the enolate. This increase in stability should therefore increase the acidity of the glycine protons. However, the impact of these groups on the reactivity of the complexes is more complicated than just the considerations given to p*K*_a_, especially in the case of the trifluoromethylated version. Central to these concerns is the effect of increased steric bulk by these moieties on the area surrounding the active site of the complex. Therefore, in order to fully understand the impact of the incorporation of these groups, one must understand the effects that they will have on the structure as well as the impact that their incorporation will have on the electronic nature of the complex and its p*K*_a_. With this in mind, a complex incorporating a 2-aminoacetophenone moiety was also introduced in the study to evaluate the effect of the phenyl groups of the 2-aminobenzophenones with respect to steric concerns.

**Figure 2:**
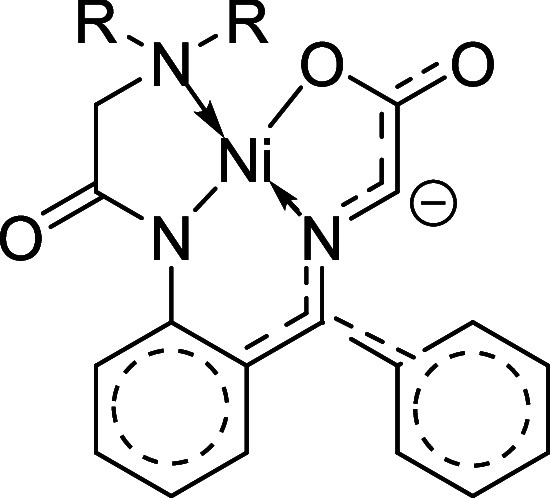
Resonance hybrid system of Ni­(II) glycine Schiff’s base enolate.

## Results and Discussion

Therefore, a series of known and new Ni­(II) complexes of glycine Schiff bases were prepared, purified, and crystallized to obtain a full set of characterization. (Scheme 1) The primary deviation of the synthesis of these systems was the application of the substituted 2-aminobenzo/acetophenone **1a**–**d** that was employed. While most of the 2-aminobenzo/acetophenones were commercially available, the 2-[4-(trifluoromethyl)­benzoyl]­analine **1d** was prepared from the reaction of 2-aminobenzonitrile and two equiv of the freshly prepared Grignard Reagent of either 4-(trifluoromethyl)-bromobenzene or 3,5-bis­(trifluoromethyl)-bromobenzene. Subsequently, each of the amino-benzo/acetophenones **1a**–**d** were treated with bromoacetyl bromide in acetonitrile with potassium carbonate as the base. The 2-bromoacetamides **2a**–**d** that resulted were treated with dibutyl amine in acetonitrile with excess potassium carbonate to quench the liberated proton resulting in the formation of the appropriate ligands **3a**–**d**. The Schiff base formation and metal complexation of each of these ligands was accomplished by treating the ligands **3a**–**d** with glycine and nickel­(II) nitrate hexahydrate in a basic methanolic solution. Following the standard workup procedure, the red crystalline complexes **4a**–**d** were isolated by filtration, purified by flash silica chromatography (dichloromethane:acetone), and characterized by ^1^H NMR, ^13^C NMR, HRMS, and melting point when applicable.

**Scheme 1:**
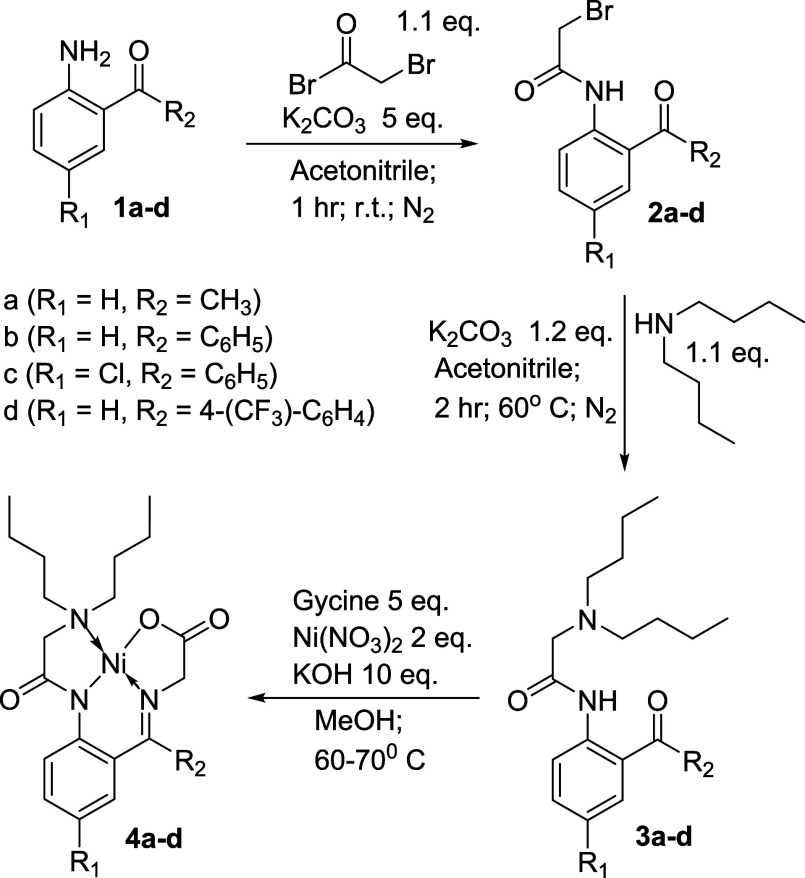
Synthesis of Ni­(II) Glycine Schiff Bases **4a**–**d**

With access to the glycine derivatives, the analysis of their reactivity became the focus of subsequent studies. Initially, a series of competitive reactivity experiments were conducted in which an equimolar mixture of two complexes was treated with a limited quantity of benzyl bromide in a biphasic combination of dichloromethane and 30% aqueous potassium hydroxide with tetrabutylammonium bromide as the catalyst (Scheme 2). Therefore, three experiments were conducted comparing complexes **4a**,**c**-**d** to the unsubstituted 2-aminobenzophenone derived complex **4b** (Table 1). In the first experiment, the reactivity of the acetophenone derived complex **4a** was compared to complex **4b**. This experiment demonstrated that the impact of the electronic effects afforded by the phenyl group slightly outweighed the decrease in crowding afforded by the methyl group, as **4b** was favored in a 59:41 ratio. Next the impact of electron withdrawing groups was evaluated by comparing the reactivities of complexes **4c**-**d**. The incorporation of both the chloride on the rotationally locked phenyl group in **4c** and the trifluoromethyl group on the less rotationally restricted phenyl group in **4d** resulted in an increased reactivity of the complexes. The incorporation of either of these groups resulted in a drastic increase in reactivity as the chlorinated complex **4c** resulted in a 10-fold reactivity difference compared to the unsubstituted **4b**, producing the benzylated product **5c** in a 91:9 ratio compared to **5b**. While in the case of the trifluoromethyl containing complex **4d**, the benzylated product **5b** was not observed in the crude reaction mixture resulting in the exclusive formation of **5d**.

**Scheme 2:**
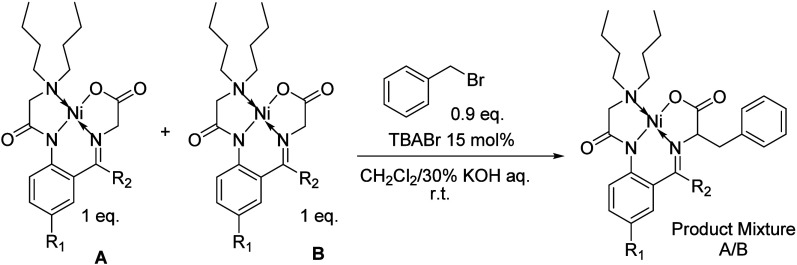
Competitive Benzylation of Ni­(II) Complexes **4a**–**d** to Produce the Phenylalanine Derived Products **5a**–**d** under Phase Transfer Conditions

**Table 1:** Rate of Benzylation Comparison among Complexes **4a–d**

				Product A			Product B			Mol %
Experiment	Complex A	Complex B	Time	R_1_		R_2_	R_1_		R_2_	Product A/B
1	**4b**	**4a**	1 h	**5b**	H	Ph	**5a**	H	CH_3_	59/41
2	**4b**	**4c**	1 h	**5b**	H	Ph	**5c**	Cl	Ph	9/91
3	**4b**	**4d**	1 h	**5b**	H	Ph	**5d**	H	4-CF_3_Ph	>1/<99

To further define the reactivity characteristics of these complexes, our attention turned to the bis-alkylation of the glycine unit to compare the reactivity of the complexes versus the electronic and steric nature surrounding the reactive glycine methylene moiety. The conditions selected for these investigations were the application of propargyl bromide under phase transfer conditions employing dichloromethane and 30% aqueous potassium hydroxide with tetrabutylammonium bromide as the catalyst. This system was selected because of previous experience with Ni­(II) complexes of glycine Schiff bases in which bis-propargylation was achieved with limited success and required relatively long reaction times (24 h) providing room for improvement and analysis. This is in comparison to other primary alkyl halides, which provide monoalkylation under phase transfer conditions and bis-alkylation under homogeneous reaction conditions with potassium hydroxide in DMF and require less than 15 min to complete.

In this case, competitive reactions were not necessary to generate a reactivity profile, as the relatively long reaction times required for the procedure offered ample opportunity to evaluate the process by decreasing the reaction time. Therefore, the standard conditions that were applied for the propargylation of the Ni­(II) complexed glycine Schiff bases **4a**-**d** were 3.5 equiv of propargyl bromide in a biphasic solution of dichloromethane a 30% (w/v) aqueous solution of potassium hydroxide with tetrabutylammonium bromide as the catalyst (Scheme 3). The reactions were conducted under a nitrogen atmosphere with vigorous stirring (∼750 rpm) at room temperature for 12 h. In each case, the crude reaction produced a mixture of products **6a**-**d** and **7a**-**d** without any of the starting materials **4a**-**d** observed.

**Scheme 3:**
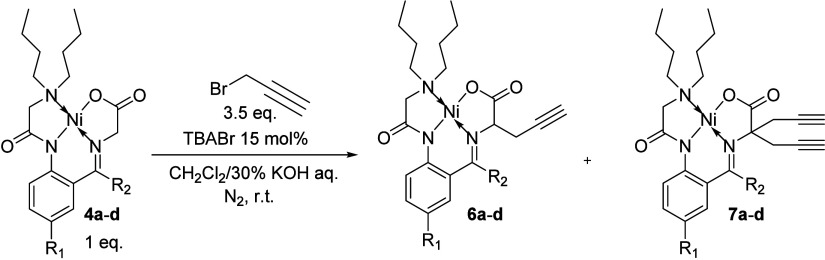
Propargylation of Ni­(II) Complexes **4a**–**d** with Propargyl Bromide

The propargylation of the acetophenone derived **4a** yielded only 15% of the bis-propargyl product **7a**, 65% of **6a**, and 20% of an additional product (Table 2). This product proved to be difficult to isolate; however, the results of mass spectroscopy and proton NMR show that the mixture is a combination of bis-propargylated products. These products result from either the bis-alkylation of the glycine moiety or from the monoproparglyation of the glycine unit combined with the monopropargylation of the methyl group of the acetophenone moiety and the methylene moiety of the protected glycine. Therefore, it was expected that the decreased steric constraint offered by complex **4a** for the synthesis of sterically constrained bis-alkylated glycine derivatives provides complications due to competing enolate structures. The application of the unsubstituted benzophenone derived complex **4b** resulted in only 11% conversion to the bis-propargylated product. The application of the chlorine substituted derivative **4c** yielded products **6c** and **7c** in a 54:46 ratio. This indicates that, again, the application of electron withdrawing substituents in the extended enolate system results in a drastic increase in reactivity. However, the application of the trifluoromethyl substituted complex **4d** only yielded a 33% conversion to the corresponding bis-propargylated product **7d**. While this conversion was slightly lower than expected from the results provided under the competitive monobenzylation studies described earlier, this may have been the result of the added steric and electrostatic interactions of this system.

**Table 2:** Rates of the Propargylation of Complexes **4a**–**d**

Experiment	R_1_	R_2_	Time (H)	Mol Percent Products **6**/**7**
1^a^	CH_3_	H	12	65:15
2	Ph	H	12	89:11
3	Ph	Cl	12	54:46
4	4-CF_3_-Ph	H	12	67:33

a A third product resulting from the propargylation of the methyl group R_1_ of the acetophenone moiety accounted for the remaining 20 mol %.

## Conclusion

In conclusion, the electronic stability offered via the incorporation of a phenyl moiety in the benzophenone derived series of Ni­(II) complexes of glycine **4b**-**d** outweighs the steric availability of the reactive site afforded by the acetophenone derived complex **4a** under conditions suitable for simple monoalkylation of the reactive glycine. Additionally, the incorporation of an electron withdrawing group within the extended enolate system of these complexes yields increased reactivity, presumably through their stabilization of the enolate intermediate. The reactivity offered through this stability extends to the reactivity of these metal complexed systems to produce more sterically constrained species such as the bis-propargylated products **7b**-**d**. However, the steric effects and potential electrostatic interactions seem to play a larger role in the process associated with second alkylation. Additionally, the Ni­(II) complex, **4c**, which was first described within this paper reacted at the highest rate for the bis-propargylation experiments.

## Methods

^1^H and ^13^C NMR spectra were performed on a Brüker Advance 300 spectrometer using TMS and CDCl_3_ as internal standards. High Resolution Mass Spectra (HRMS) were recorded on a Agilent 6545 high-mass-resolution Q-TOF mass spectrometer. Melting points (M.p.) are uncorrected and were obtained in open capillaries. All reagents and solvents, unless otherwise stated, are commercially available and were used as received. Unless otherwise stated, yields refer to isolated yields of products of >95% purity as estimated by ^1^H and ^13^C NMR spectrometry. All new compounds were characterized by ^1^H, ^13^C NMR, and HRMS.

### Preparation of 2-[4-(Trifluoromethyl)­benzoyl]­aniline **1d**. General Procedure

To activated magnesium turnings (3.0 equiv) in dry THF was slowly added the trifluoromethylated bromobenzene (2.1 equiv). Following the complete addition of bromobenzene, the reaction was catalyzed by heat until a brown color was observed, and the reaction began to reflux on its own. After the exothermic reaction concluded, the reaction was refluxed for 30 min. The reaction was allowed to cool to room temperature before the dropwise addition of 2-aminobenzonitrile (0.95 equiv) in THF. Following the complete addition, the reaction was allowed to reflux overnight with stirring. The reaction was cooled, slowly quenched with water followed by 3 N HCl, and refluxed for 12 h. The resulting solution was carefully neutralized with concentrated sodium bicarbonate. The crude product was obtained by extraction with methylene chloride. The final purified product **1d** was obtained by column chromatography with ethyl acetate and hexane as the mobile phase.

### 2-[4-(Trifluoromethyl)­benzoyl]­aniline **1d**

Mp 101.3 °C. ^1^H NMR δ 6.13 (2H, bs), 6.52 (1H, m), 6.67 (1H, d, *J* = 8.3 Hz), 7.23 (2H, m), 7.64 (4H, m). 13C NMR δ: 115.7, 117.2, 117.3, 125.2, 127.4 (1C, q, *J* = 270.8 Hz), 129.1, 132.5 (1C, q, *J* = 32.4 Hz), 134.4, 134.9, 143.5, 151.3, 197.7. HRMS [M+H^+^] found *m*/*z* 266.0919, calcd for C_14_H_11_F_3_NO 266.0787.

### Condensation of 2-Aminobenzo/acetophenone **1a**–**d** and Bromoacetyl Bromide Yielding *N*-(2-Benzoyl/acetyl-phenyl)-2-bromo-acetamides **2a**–**d**. General Procedure

A solution of bromoacetyl bromide (1.1 equiv) in acetonitrile (2 mL/1 g of **1a**–**d**) was slowly added to a slurry of 2-aminobenzo/acetophenone **1a**–**d** (1 equiv) and potassium carbonate (5 equiv) in acetonitrile (3.5 mL/1 g K_2_CO_3_). The reaction was stirred at ambient temperature (room temperature water bath) for 1 h, and upon completion (monitored by TLC), the acetonitrile was evaporated under vacuum. Water was then added to the crude mixture and extracted with dichloromethane three times. The organic portions were combined, dried, and concentrated under vacuum to afford the corresponding α-bromoamide product **2a**–**e** in 98% yield and greater than 99% chemical purity.

### *N*-(2-Acetylphenyl)-2-bromoacetamide **2a**

Mp 75.1 °C. ^1^H NMR δ 2.69 (3H, s), 4.19 (2H, s), 7.19 (1H, m), 7.59 (1H, m), 7.93 (1H, dd, *J* = 7.91, 1.61 Hz), 8.73 (1H, dd, *J* = 8.50, 1.17 Hz), 12.5 (1H, bs). ^13^C NMR δ 28.5, 43.2, 120.6, 122.3, 123.2, 131.4, 134.8, 139.5, 165.5, 202.2. HRMS [M+Na^+^] found *m*/*z* 277.9784, calcd for C_10_H_10_BrNNaO_2_ 277.9793.

### *N*-(2-Benzoyl-phenyl)-2-bromo-acetamide **2b**

Mp 71.7 °C. ^1^H NMR δ 4.20 (2H, s), 7.15 (1H, m), 7.43–7.53 (2H, m), 7.53–7.63 (3H, m), 7.63–7.75 (2H, m), 8.61 (1H, dd, *J* = 8.80, 1.17 Hz), 11.6 (1H, bs). ^13^C NMR δ 43.0, 121.3, 122.9, 124.0, 128.1, 129.8, 132.4, 133.3, 133.9, 138.1, 139.0, 165.1, 198.8. HRMS [M+Na^+^] found *m*/*z* 339.9955, calcd for C_15_H_12_BrNNaO_2_ 339.9949.

### *N*-(2-Benzyol-4-chlorophenyl)-2-bromoacetamide **2c**

Mp 84.9 °C. ^1^H NMR δ 4.05 (2H, s), 7.53–7.61 (4H, m), 7.64 (1H, tt, *J* = 7.5, 1.5 Hz), 7.75–7.79 (2H, m), 8.61 (1H, d, *J* = 9.9 Hz). ^13^C NMR δ 29.4, 123.1, 125.5, 128.4, 128.7, 130.1, 132.7, 133.2, 133.9, 137.6, 137.9, 165.0, 197.9. HRMS [M+Na^+^] found *m*/*z* 373.9427, calcd for C_16_H_12_BrClNNaO_2_ 372.9601.

### *N*-(2-(4-(1,1,1-Trifluoromethyl)-benzoyl-phenyl)-2-bromoacetamide **2d**

Mp 67.3 °C. ^1^H NMR δ 4.03 (2H, s), 7.17 (1H, td, *J* = 8.1, 0.6 Hz), 7.54 (1H, dd, *J* = 8.1, 1.8 Hz), 7.65 (1H, td, *J* = 8.4, 1.2 Hz), 7.75–7.88 (4H, m), 8.69 (1H, dd, *J* = 8.1, 0.6 Hz), 11.50 (1H, s). ^13^C NMR δ 121.8, 123.2, 123.3, 125.4, 125.5, 125.6, 130.1, 130.3, 133.5, 134.0 (1 C, q, *J* = 32.85 Hz), 134.9, 139.9, 141.6, 165.1, 198.2. ^19^F NMR δ −63.08. HRMS [M+Na^+^] found *m*/*z* 407.9932, calcd for C_16_H_11_BrF_3_NNaO_2_ 407.9817.

### Alkylation of Dibutylamine with *N*-(2-Benzyoly/acetyl-phenyl)-2-bromo-acetamides **2a**–**d**, Yielding the Corresponding *N*-(2-Benzoyl/acetyl-phenyl)-2-Dibutyllamino-acetamides **3a**–**d**. General Procedure

To a slurry of *N*-(2-benzoyl/acetyl-phenyl)-2-bromo-acetamide **2a**–**d** (1 equiv) and potassium carbonate (1.2 equiv) in acetonitrile (10 mL/1g of **2a**–**d**) was added the corresponding dibutylamine (1.1 equiv). The reaction was allowed to proceed for 2 h at 60–70 °C (monitored by TLC) before the reaction mixture was concentrated under vacuum. Water was added to the viscous liquid, followed by extraction with dichloromethane. The organic portions were combined, dried with magnesium sulfate, and concentrated in a vacuum to afford the corresponding *N*-(2-benzoyl/acetyl-phenyl)-2-dibutylamino-acetamides **3a**–**d** in nearly quantitative yield and high chemical purity >99%.

### *N*-(2-Acetyl-phenyl)-2-dibutylamino-acetamide **3a**

^1^H NMR δ 0.89 (3H, t, *J* = 7.2 Hz), 1.32 (4H, s, *J* = 7.2 Hz), 1.48–1.58 (4H, m), 2.55–2.64 (4H, m), 2.65 (3H, s), 3.22 (2H, s), 7.12 (1H, td, *J* = 8.4, 1.2 Hz), 7.54 (1H, td, *J* = 8.7, 1.5 Hz), 7.88 (1H, dd, *J* = 8.4, 1.5 Hz), 8.84 (1H, dd, *J* = 8.4, 1.2 Hz), 12.42 (1H, s). ^13^C NMR δ 14.1, 20.1, 28.6, 29.2, 55.5, 59.9, 121.0, 122.4, 123.2, 131.3, 134.5, 139.9, 172.8, 201.1. HRMS [M+Na^+^] found *m*/*z* 327.2008, calcd for C_18_H_28_N_2_NaO_2_ 327.2048.

### *N*-(2-Benzoyl-phenyl)-2-dibutylamino-acetamide **3b**

^1^H NMR δ 0.78 (6H, t, *J* = 7.5 Hz), 1.25 (4H, s, *J* = 7.5 Hz), 1.46 (4H, p, *J* = 7.5 Hz), 2.51 (4H, t, *J* = 7.5 Hz), 3.17 (2H, s), 7.09 (1H, dt, *J* = 8.1, 1.2 Hz), 7.45–7.61 (4H, m), 7.77 (1H, d, *J* = 8.7 Hz), 8.63 (1H, d, *J* = 8.7 Hz), 11.38 (1H, bs). ^13^C NMR δ 14.2, 20.8, 29.5, 55.7, 59.9, 122.0, 122.5, 126.1, 128.5, 130.3, 132.3, 132.8, 133.3, 138.6, 139.0, 172.3, 197.9. HRMS [M+H^+^] found *m*/*z* 367.2251, calcd for C_23_H_31_N_2_O_2_ 367.2380.

### *N*-(2-Benzoyl-4-chlorophenyl)-2-dibutylamino-acetamide **3c**

^1^H NMR δ 0.80 (3H, t, *J* = 1.5 Hz), 1.16 (4H, s, *J* = 7.5 Hz), 1.41–1.51 (4H, m), 2.52 (4H, t, *J* = 7.5 Hz), 3.18 (2H, s), 7.44 (1H, d, *J* = 2.7 Hz), 7.49–7.54 (3H, m), 7.63 (1H, t, *J* = 7.2 Hz), 7.79 (2H, dd, *J* = 6.9, 1.8 Hz), 8.63 (1H, d, *J* = 8.7 Hz), 11.29 (1H, s). ^13^C NMR δ 14.0, 20.5, 29.3, 55.4, 59.6, 123.2, 127.2, 127.4, 128.5, 130.1, 131.2, 132.8, 133.1, 137.2, 137.6, 172.0, 196.3. HRMS [M+Na^+^] found *m*/*z* 423.1559, calcd for C_23_H_29_ClN_2_NaO_2_ 423.1815.

### *N*-(2-(4-Trifluoromethyl-benzoyl)-phenyl)-2-dibutylamino-acetamide **3d**

^1^H NMR δ 0.83 (3H, t, *J* = 7.2 Hz), 1.29 (4H, s, *J* = 7.2 Hz), 1.45–1.55 (4H, m), 2.56 (4H, t, *J* = 7.2 Hz), 3.22 (2H, s), 7.13 (1H, td, *J* = 7.5, 0.6 Hz), 7.46 (1H, dd, *J* = 7.5, 1.8 Hz), 7.61 (1H, td, *J* = 8.4, 1.5 Hz), 7.77 (2H, d, *J* = 8.4 Hz), 7.89 (2H, d, *J* = 8.7 Hz), 8.69 (1H, d, *J* = 8.7 Hz), 11.56 (1H, s). ^13^C NMR δ 14.0, 20.5, 55.4, 59.7, 121.9, 122.4, 125.1, 125.3, 125.4, 125.5, 130.1, 132.3, 133.8 (1 C, q, *J* = 32.3 Hz), 134.0, 139.3, 141.7, 172.2, 196.6. HRMS [M+Na^+^] found *m*/*z* 457.2078, calcd for C_24_H_29_F_3_N_2_NaO_2_ 457.2073.

### Synthesis of the Ni­(II) Complexes of Glycine Schiff Bases with *N*-(2-Benzyoly/acetyl-phenyl)-2-Dibutylamino-acetamides **3a**–**d**. General Procedure

A solution of potassium hydroxide (10 equiv) in methanol (7 mL/1 g of KOH) was added to a suspension of *N*-(2-benzyoly/acetyl-phenyl)-2-dibutylamino-acetamides **3a**–**d** (1 equiv), glycine (5 equiv), and nickel nitrate hexahydrate (2 equiv) in methanol (10 mL/1 g of **3a**–**d**) at 60–70 °C. Upon complete consumption of the *N*-(2-benzyoly/acetyl-phenyl)-2-dibutylamino-acetamides **3a**–**d**, monitored by TLC, the reaction mixture was poured over a slurry of ice and 5% acetic acid. After the complete precipitation, product **4a**–**d** was filtered and dried, in a low-temperature oven (50 °C) overnight. The product was obtained in high chemical yield (>90%). The Ni (II) complexes were purified via flash silica chromatography with dichloromethane (CH_2_Cl_2_) and acetone as the eluents.

### Ni­(II) Complex of Glycine Schiff Base with *N*-(2-Acetyl-phenyl)-2-dibutylamino-acetamide **4a**

Mp 200.2 °C. ^1^H NMR δ 1.05 (6H, t, *J* = 6.87 Hz), 1.51 (4H, m), 2.28 (4H, m), 2.41 (3H, s), 2.78 (2H, m), 2.92 (2H, m), 3.33 (2H, s), 4.13 (2H, s), 7.02 (1H, m), 7.37 (1H, m), 7.61 (1H, d, *J* = 7.8 Hz), 8.46 (1H, d, *J* = 8.2 Hz). ^13^C NMR δ 14.0, 19.1, 20.8, 28.9, 59.9, 60.0, 62.0, 121.7, 124.8, 126.4, 129.3, 132.1, 141.6, 169.5, 177.1, 177.9. HRMS [MH^+^] found *m/s* 418.1642, calcd for C_20_H_30_N_3_NiO_3_ 418.1635.

### Ni­(II) Complex of Glycine Schiff Base with *N*-(2-benzyoyl-phenyl)-2-dibutylamino-acetamide **4b**

Mp 174.9 °C. ^1^H NMR δ 1.08 (6H, t, *J* = 7.2 Hz), 1.44–1.64 (4H, m), 2.17–2.37 (4H, m), 2.86 (2H, dt, *J* = 12.6, 4.5 Hz), 3.04–3.17 (2H, m), 3.38 (2H, s), 3.74 (2H, m), 6.80 (1H, m), 6.90 (1H, dd, *J* = 8.1, 1.8 Hz), 7.08–7.11 (2H, m), 7.36 (1H, m), 7.51–7.57 (3H, m), 8.70 (1H, d, *J* = 8.4 Hz). ^13^C NMR δ 14.3, 21.0, 29.3, 60.5, 61.5, 62.2, 121.4, 124.5, 125.5, 126.2, 129.8, 132.8, 133.8, 135.0, 142.8, 172.0, 177.5, 179.0. HRMS [M+H^+^] found *m/s* 480.1779, calcd for C_25_H_32_N_3_NiO_3_ 480.1792.

### Ni­(II) Complex of Glycine Schiff Base with *N*-(2-Benzyoyl-4-chlorophenyl)-2-dibutylamino-acetamide **4c**

Mp 218.6 °C. ^1^H NMR δ 1.09 (6H, t, *J* = 7.5 Hz), 1.48–1.63 (4H, m), 2.20–2.36 (4H, m), 2.89 (2H, td, *J* = 12.0, 4.5 Hz), 3.12–3.18 (2H, m), 3.36 (2H, s), 3.74 (2H, s), 6.86 (1H, d, *J* = 2.7 Hz), 7.08–7.11 (2H, m), 7.30 (1H, td, *J* = 7.5, 2.7 Hz), 7.57–7.59 (3H, m), 8.73 (1H, d, *J* = 9.0 Hz). ^13^C NMR δ: 14.0, 20.8, 29.2, 60.4, 61.5, 61.8, 125.6, 125.8, 125.9, 126.5, 129.9, 130.0, 132.2, 132.4, 134.1, 141.4, 171.1, 177.0, 179.0. HRMS [M+H^+^] found *m/s* 514.1397, calcd for C_25_H_31_ClN_3_NiO_3_ 514.1402.

### Ni­(II) Complex of Glycine Schiff Base with *N*-(2-(4-1,1,1-trifluoromethyl)-benzoyl-phenyl)-2-dibutylamino-acetamide **4d**

Mp 167.2 °C. ^1^H NMR δ 1.09 (6H, t, *J* = 7.5 Hz), 1.55 (4H, s, *J* = 7.5 Hz), 2.22–2.38 (4H, m), 2.85–2.93 (2H, m), 3.11–3.17 (2H, m), 3.39 (2H, s), 3.70 (2H, s), 6.82 (2H, m), 7.29 (2H, d, *J* = 7.2 Hz), 7.39 (1H, m), 7.85 (2H, d, *J* = 8.1 Hz), 8.73 (1H, d, *J* = 8.7 Hz). ^13^C NMR δ 121.3, 124.5, 124.6, 126.7, 127.0 (1 C, q, *J* = 272.0 Hz), 131.9, 133.2, 138.3, 142.9, 170.1, 176.8, 179.0. ^19^F NMR δ −161.7. HRMS [M+Na^+^] found *m/s* 570.1329, calcd for C_26_H_30_F_3_N_3_NaO_3_ 570.1485.

### Competitive Phase Transfer Benzylation or Methylation of Ni­(II) Complexes of Glycine Schiff Bases **5a**–**c**

A stock solution (approximately 0.06 M) was prepared for complexes **4a**–**d** in CDCl_3_ (1–2 mL). A mixture of two complexes for comparison was prepared by transferring equal amounts of each complex (500 μL) to an NMR tube. ^1^H NMR spectroscopy was performed to ensure that the complexes were equimolar. Adjustments were made through appropriate quantities of the necessary solution until the mixture was equimolar. The contents were transferred from the NMR tube into a appropriately sized reaction vessel and the solvent was removed under vacuum. Separate solutions of benzyl bromide (0.027 M) and tetrabutylammonium bromide (0.0090 M) in CH_2_Cl_2_ were prepared. An appropriate amount of each of these solutions (∼1 mL each) was transferred to the dry equimolar complex mixture to achieve final solution that contains the alkylating reagent (0.9 equiv), 15 mol % of the tetrabutyl ammonium bromide, and 1 equiv of each complex. The reaction vessel was purged with N_2_, and approximately 2 mL of 30% aqueous potassium hydroxide was added. The reactions were allowed to react at room temperature with vigorous stirring (∼750 rpm) for 1 h. Additional amounts of water (10 mL) and CH_2_Cl_2_ (5 mL) were added. After diluting the reaction mixture, the organic fraction is extracted, and this procedure was repeated three times. The organic fractions were combined, dried with MgSO_4_, filtered, and evaporated under vacuum which yielded the crude reaction mixture for further ^1^H NMR analysis.

### Phase Transfer Alkylation of Ni­(II) Complexes of Glycine Schiff Base with *N*-(2-Benzoyl/acetyl-phenyl)-2-Dibutylamino-Acetamide **4a**–**d** with Benzyl Bromide for Reference. General Procedure

To a solution of **4a**–**d** in CH_2_Cl_2_ (1 mL/g) at room temperature was added tetrabutylammonium bromide (0.15 equiv), 30% sodium hydroxide solution (1 mL/mL CH_2_Cl_2_), and benzyl bromide (1.2 equiv). The resultant mixture was rigorously stirred (∼750 rpm) at room temperature under nitrogen for 1 h. An additional aliquot of water and CH_2_Cl_2_ were added to dilute the mixture, and the water was extracted several times with CH_2_Cl_2_. The organic layer was dried with MgSO_4_, filtered, and evaporated in vacuum to yield crystalline compounds **5a**–**d**. These complexes were purified by silica gel chromatography using CH_2_Cl_2_ and acetone as the eluent.

### Ni­(II) Complex of Phenylalanine Schiff Base with *N*-(2-Acetyl-phenyl)-2-dibutylamino-acetamide **5a**

Mp 265.1 °C. ^1^H NMR δ 0.96 (3H, t, *J* = 7.5 Hz), 0.97 (3H, t, *J* = 7.5 Hz), 1.25 (2H, s, *J* = 7.2 Hz), 1.30–1.55 (4H, m), 1.95–2.06 (2H, m), 2.08–2.22 (2H, m), 2.40 (3H, s), 2.42 (1H, m), 2.61 (1H, m), 2.82 (1H, AB, *J* = 15.6 Hz), 3.25 (1H, ABX, *J* = 13.5, 4.5 Hz), 3.42 (1H, AB, *J* = 15.6 Hz), 3.63 (1H, ABX, *J* = 13.5, 4.5 Hz), 4.45 (1H, t, *J* = 4.5 Hz), 7.04 (1H, m), 7.38 (1H, m), 7.46–7.49 (2H, m), 7.53–7.57 (3H, m), 7.67 (1H, dd, *J* = 8.1, 1.8 Hz), 8.44 (1H, dd, *J* = 8.7, 1.2 Hz). ^13^C NMR δ 13.9, 14.1, 18.1, 20.5, 20.8, 24.1, 29.4, 40.1, 55.3, 58.1, 63.6, 71.5, 121.6, 124.1, 126.6, 127.7, 129.0, 129.5, 131.5, 132.3, 133.0, 136.2, 141.8, 165.1, 167.7, 175.9, 178.1. HRMS [M+Na^+^] found *m*/*z* 781.2156, calcd for C_27_H_35_N_3_NaNiO_3_ 530.1924.

### Ni­(II) Complex of Phenylalanine Schiff Base with *N*-(2-benzoyl-phenyl)-2-dibutylamino-acetamide **5b**

Mp 159.7 °C. ^1^H NMR δ 0.92 (3H, t, *J* = 5.7 Hz), 0.98 (3H, t, *J* = 5.7 Hz), 1.16–1.31 (4H, m), 1.41 (1H, s, *J* = 2.4 Hz), 1.52 (1H, s, *J* = 2.4 Hz), 1.89–2.00 (2H, m), 2.08–2.19 (2H, m), 2.69 (1H, m), 2.75 (1H, ABX, *J* = 10.2, 3.9 Hz), 2.81 (1H, AB, *J* = 12.0 Hz), 3.04 (1H, ABX, *J* = 10.2, 3.9 Hz), 3.38 (1H, AB, *J* = 12.0 Hz), 4.12 (1H, q, *J* = 5.4 Hz), 6.79 (2H, d, *J* = 2.7 Hz), 7.05 (1H, dd, *J* = 5.4, 0.6 Hz), 7.30–7.33 (2H, m), 7.43–7.45 (2H, m), 7.51–7.57 (6H, m), 8.45 (1H, dd, *J* = 6.6, 0.6 Hz). ^13^C NMR δ: 13.9, 14.1, 20.4, 20.8, 23.5, 29.4, 39.3, 54.9, 57.9, 63.7, 71.4, 121.2, 123.7, 127.0, 127.3, 127.7, 128.9, 129.1, 129.2, 130.0, 131.5, 132.8, 133.8, 136.3, 142.8, 170.7, 176.0, 178.1. HRMS [M+Na^+^] found *m*/*z* 592.2239, calcd for C_32_H_37_N_3_NaNiO_3_ 592.2080.

### Ni­(II) Complex of Phenylalanine Schiff Base with *N*-(2-Benzoyl-4-chlorophenyl)-2-dibutylamino-acetamide **5c**

Mp 171.4 °C. ^1^H NMR δ 0.93 (3H, t, *J* = 6.9 Hz), 0.99 (3H, t, *J* = 6.9 Hz), 1.21 (4H, m), 1.46 (2H, m), 1.95 (2H, m), 2.12 (2H, m), 2.43 (1H, m), 2.66 (2H, m), 2.83­(1H, m), 3.05 (1H, m), 3.37 (1H, AB, *J* = 16.0 Hz), 4.12 (1H, m), 6.73 (1H, m), 7.04 (1H, m), 7.26 (2H, m), 7.41 (2H, m), 7.56 (6H, m), 8.45 (1H, d, *J* = 9.2 Hz). ^13^C NMR δ 13.9, 14.0, 20.4, 20.8, 23.6, 29.4, 39.3, 55.1, 58.1, 63.5, 71.7, 125.0, 125.9, 127.2, 127.5, 127.7, 128.2, 128.9, 129.3, 129.5, 130.4, 131.4, 132.4, 132.6, 133.1, 136.1, 141.4, 170.1, 176.2, 177.9. HRMS [M+Na^+^] found *m*/*z* 626.1713, calcd for C_32_H_36_ClN_3_NaNiO_3_ 626.1696.

### Ni­(II) Complex of Phenylalanine Schiff Base with *N*-(2-(4-Trifluoromethylbenzoyl)-phenyl)-2-dibutylamino-acetamide **5d**

Mp 207.6 °C. ^1^H NMR δ 0.93 (3H, t, *J* = 5.4 Hz), 0.98 (3H, t, *J* = 5.4 Hz), 1.17–1.54 (6H, m), 1.94–2.02 (2H, m), 2.11–2.20 (2H, m), 2.48 (1H, td, *J* = 9.3, 3.3 Hz), 2.71 (1H, m), 2.72 (1H, ABX, *J* = 10.2, 3.9 Hz), 2.83 (1H, AB, *J* = 12.0 Hz), 3.13 (1H, ABX, *J* = 10.2, 3.9 Hz), 3.42 (1H, AB, *J* = 12.0 Hz), 4.11 (1H, m), 6.67 (1H, dd, *J* = 6.3, 1.2 Hz), 6.81 (1H, td, *J* = 6.3, 0.9 Hz), 7.13 (1H, d, *J* = 6.0 Hz), 7.35 (1H, td, *J* = 5.4, 1.5 Hz), 7.40–7.43 (2H, m), 7.49 (1H, d, *J* = 6.3 Hz), 7.54–7.58 (3H, m), 7.77 (1H, d, *J* = 6.0 Hz), 7.84 (1H, d, *J* = 6.0 Hz), 8.49 (1H, dd, *J* = 5.4, 0.9 Hz). ^13^C NMR δ 13.9, 14.0, 20.4, 20.8, 23.8, 29.4, 29.7, 55.1, 58.2, 63.6, 71.6, 121.3, 124.0, 125.2, 126.2, 126.3, 126.4, 127.8, 128.0, 128.3, 129.0, 131.4, 132.0 (1C, q, *J* = 32.9 Hz), 133.2, 133.3, 135.9, 137.3, 137.4, 143.1, 169.0, 176.3, 177.7. HRMS [M + H+] found *m*/*z* 638.2449, calcd for C_33_H_36_F_3_N_3_NiO_3_ 638.2135.

### Propargylation of Ni­(II) Complexes of Glycine Schiff Base with *N*-(2-Benzoyl/acetyl-phenyl)-2-dibutylamino-acetamide **4a**–**d** with Propargyl Bromide under Phase Transfer Conditions. General Procedure

To a solution of **4a**–**d** in CH_2_Cl_2_ (1 mL/g) at room temperature was added tetrabutylammonium bromide (0.15 equiv), 30% sodium hydroxide solution (1 mL/mL CH_2_Cl_2_), and propargyl bromide (3.5 equiv). The resultant mixture was rigorously stirred (∼750 rpm) at room temperature under nitrogen for 12 h. An additional aliquot of water and CH_2_Cl_2_ were added to dilute the mixture, and the water was extracted several times with CH_2_Cl_2_. The organic layer was dried with MgSO_4_, filtered, and evaporated under vacuum to yield crystalline products. The crude reaction mixture was analyzed via ^1^HNMR and then the materials were purified with silica gel chromatography using CH_2_Cl_2_ and acetone as the eluent to yield pure materials of **6a**–**d**, and **7a**–**d**.

### Ni­(II) Complex of 2-Amino-pent-4-ynoic Acid Schiff Base with *N*-(2-Acetyl-phenyl)-2-dibutylamino-acetamide **6a**

Mp 225.6 °C. ^1^H NMR δ 1.00 (6H, t, J = 7.3 Hz), 1.25–1.54 (4H, m), 1.90 (1H, m), 2.21 (1H, m), 2.17–2.63 (7H, m), 2.47 (3H, s), 2.85 (1H, m), 3.00 (2H, m), 3.26 (1H, m), 3.83 (1H, ABX, J = 16.3 Hz), 4.25 (1H, t, J = 5.0 Hz), 7.00 (1H, m), 7.35 (1H, m), 7.65 (1H, d, J = 8.2 Hz), 8.49 (1H, d, J = 8.6 Hz). ^13^C NMR δ 13.9, 14.0, 18.8, 20.7, 20.8, 24.9, 27.1, 29.2, 57.5, 60.2, 62.8, 67.7, 74.0, 79.1, 121.5, 124.2, 126.4, 129.6, 132.4, 141.9, 169.2, 176.8, 177.9. HRMS [M+H^+^] found *m*/*z* 456.1790, calcd for C_23_H_32_N_3_NiO_3_ 456.1792.

### Ni­(II) Complex of 2-Amino-pent-4-ynoic Acid Schiff Base with *N*-(2-Benzoyl-phenyl)-2-dibutylamino-acetamide **6b**

Mp 112.5 °C. ^1^H NMR δ 1.00 (3H, t, *J* = 7.5 Hz), 1.05 (3H, t, *J* = 7.2 Hz), 1.39 (2H, p, *J* = 7.5 Hz), 1.55 (2H, p, *J* = 6.3 Hz), 1.81 (2H, m), 2.19–2.47 (4H, m), 2.51–2.74 (5H, m), 2.92 (1H, td, *J* = 8.4, 1.2 Hz), 3.03 (1H, d, *J* = 16.2 Hz), 3.98 (1H, m), 6.75–6.77 (2H, m), 7.05 (1H, m), 7.25 (1H, m), 7.33 (1H, m), 7.46–7.53 (3H, m), 8.58 (1H, d, *J* = 8.7 Hz). ^13^C NMR δ 13.9, 14.0, 20.6, 20.9, 23.7, 26.7, 29.3, 57.0, 60.5, 62.7, 67.5, 74.2, 79.6, 121.1, 123.8, 126.6, 126.7, 127.7, 129.1, 129.2, 130.0, 132.9, 133.8, 133.8, 142.9, 171.8, 177.0, 178.3. HRMS [M+H^+^] found *m*/*z* 518.1943, calcd for C_28_H_34_N_3_NiO_3_ 518.1949.

### Ni­(II) Complex of 2-Amino-pent-4-ynoic Acid Schiff Base with *N*-(2-Benzoyl-4-chlorophenyl)-2-dibutylamino-acetamide **6c**

Mp 233.1 °C. ^1^H NMR δ 1.00 (3H, t, *J* = 7.3 Hz), 1.15 (3H, t, *J* = 7.3 Hz), 1.39 (2 H, m), 1.54 (2 H, m), 1.81 (1 H, m), 2.21 (2H, m), 2.45–2.68 (7H, m), 2.92 (1H, dt, *J* = 12.3, 3.4 Hz), 3.02 (1H, ABX, *J* = 16.4 Hz), 3.90 (1H, ABX, *J* = 16.4 Hz), 4.12 (1H, m), 6.69 (1H, s), 7.04 (1H, m), 7.26 (2H, m), 7.54 (3H, m), 8.59 (1H, d, *J* = 9.2 Hz).. ^13^C NMR δ: 13.9, 14.0, 20.7, 20.9, 23.6, 26.9, 29.3, 57.3, 60.6, 62.6, 67.7, 74.3, 77.2, 79.4, 125.1, 125.8, 126.6, 127.4, 127.8, 129.4, 130.4, 132.5, 132.7, 133.1, 141.6, 171.1, 177.3, 178.1. HRMS [M+H^+^] found *m*/*z* 552.1554, calcd for C_28_H_33_ClN_3_NiO_3_ 552.1558.

### Ni­(II) Complex of 2-Amino-2-prop-2ynyl-pent-4-ynoic Acid Schiff Base with *N*-(2-(4-Trifluoromethylbenzoyl)-phenyl)-2-dibutylamino-acetamide **6d**

Mp 247.3 °C. ^1^H NMR δ 1.04­(3H,t,*J*)= 5.73 Hz), 1.05 (3H, t, *J* = 5.73 Hz), 1.41 (2H, m), 1.56 (2H, m), 1.82 (1H, m), 2.28 (2H, m), 2.49 (1H, m), 2.55–2.78 (6H, m), 2.93 (1H, dt, *J* = 8.70, 3.66 Hz), 3.05 (1H, ABX, *J* = 16.35 Hz), 3.90 (1H, m), 3.94 (1H, ABX, *J* = 16.35 Hz), 6.65 (1H, dd, *J* = 8.25, 1.47 Hz), 6.80 (1H, m), 7.25 (1H, m), 7.37 (1H, m), 7.45 (1H, m), 7.81 (2H, t, *J* = 6.3 Hz), 8.61 (1H, dd, *J* = 7.80, 0.87 Hz). ^13^C NMR δ 14.2, 14.3, 20.7, 20.9, 24.0, 26.9, 30.0, 57.2, 60.6, 62.6, 67.5, 74.5, 79.2, 121.2, 123.4 (1C, q, *J* = 270.96 Hz), 124.0, 126.0, 126.2, 127.5, 128.3, 132.3 (1C, q, *J* = 33.06 Hz), 133.3, 137.5, 143.2, 170.1, 177.1, 177.8. HRMS [M+H^+^] found *m*/*z* 586.1824, calcd for C_29_H_33_F_3_N_3_NiO_3_ 586.1822.

### Ni­(II) Complex of 2-Amino-2-prop-2-ynyl-pent-4-ynoic Acid Schiff Base with *N*-(2-Benzoyl-phenyl)-2-dibutylamino-acetamide **7b**

Mp 101.6 °C. ^1^H NMR δ 1.05 (6H, t, *J* = 7.5 Hz), 1.56 (4H, p, *J* = 7.5 Hz), 2.14 (2H, ABX, *J* = 17.1, 2.7 Hz), 2.21–2.31 (4H, m), 2.45 (2H, m), 2.66 (2H, ABX, *J* = 17.1, 2.7 Hz), 2.79–2.89 (2H, m), 3.14–3.20 (2H, m), 3.34 (2H, s), 6.67 (2H, m), 7.29 (1H, m), 7.47–7.54 (5H, m), 8.52 (1H, d, *J* = 8.4 Hz). ^13^C NMR δ 14.1, 20.8, 29.2, 30.2, 60.3, 62.1, 73.4, 76.9, 79.2, 120.8, 124.0, 127.9, 128.1, 130.0, 132.3, 134.1, 136.3, 142.4, 173.9, 178.4, 179.9. HRMS [M+H^+^] found *m*/*z* 556.2052, calcd for C_31_H_36_N_3_NiO_3_ 556.2106.

### Ni­(II) Complex of 2-Amino-2-prop-2ynyl-pent-4-ynoic Acid Schiff Base with *N*-(2-benzoyl-4-chlorophenyl)-2-dibutylamino-acetamide **7c**

Mp 166.6 °C. ^1^H NMR δ 1.05 (6H, t, *J* = 7.2 Hz), 1.54 (4H, m), 2.05–2.23 (6H, m), 2.45 (2H, s), 2.67 (2H, d, *J* = 12.2 Hz), 2.84 (2H, dt, *J* = 12.2, 5.7 Hz), 3.22 (2H, m), 3.31 (2H, s), 6.61 (1H, s), 7.25 (1H, m), 7.51 (5H, m), 8.53 (1H, d, *J* = 9.3 Hz). ^13^C NMR δ 14.1, 14.2, 20.8, 21.1, 29.3, 30.2, 60.5, 62.0, 73.5, 77.2, 77.3, 79.0, 125.3, 125.5, 127.9, 128.2, 129.2, 130.4, 132.1, 132.8, 135.6, 141.0, 173.2, 178.6, 179.8. HRMS [M+H^+^] found *m*/*z* 590.1715, calcd for C_31_H_35_ClN_3_NiO_3_ 590.1715.

### Ni­(II) Complex of 2-Amino-2-prop-2ynyl-pent-4-ynoic Acid Schiff Base with *N*-(2-(4-Trifluoromethylbenzoyl)-phenyl)-2-dibutylamino-acetamide **7d**

Mp 102.1 °C. ^1^H NMR δ 1.06­(6H, t, *J* = 7.36 Hz), 1.58 (4H, m), 2.04 (2H, dd, *J* = 17.30, 2.65 Hz), 2.27 (4H, m), 2.48 (2H, t, *J* = 2.60 Hz), 2.75 (2H, dd, *J* = 17.28, 2.64 Hz), 2.86 (2H, m), 3.19 (2H, m), 3.36 (2H, s), 6.55 (1H, dd, *J* = 8.47, 1.48 Hz), 6.74 (1H, m), 7.34 (1H, m), 7.67 (2H, m), 7.79 (2H, m), 8.55 (1H, dd, *J* = 8.65, 1.10 Hz). ^13^C NMR δ 14.1, 20.8, 29.3, 30.4, 60.4, 62.1, 73.8, 76.8, 78.8, 121.0, 123.0 (1C, q, J = 270.95 Hz), 124.3, 125.0, 127.2, 128.7, 132.3 (1C, q, J = 33.0 Hz), 132.7, 133.6, 140.1, 142.7, 172.0, 178.5, 179.6. HRMS [M+H^+^] found *m*/*z* 624.1981, calcd for C_32_H_35_F_3_N_3_NiO_3_ 624.1978.

## Supplementary Material



## Notes

^1^HNMR spectra of **1d**, **2a**–**d**, **3a**–**d**, **4a**–**d**, **5a**–**d**, **6a**–**d**, **7b**–**d** (PDF)
