# Supplementary material for: Synthesis and Evaluation of a Series of Ni(II) Complexed Nucleophilic Glycine Equivalents
Source: ACS Omega. 2025 Nov 6;10(45):54480–8. doi: 10.1021/acsomega.5c07357 (PMC12631314; doi:10.1021/acsomega.5c07357)
Supplement: Supplementary file 1 [file ao5c07357_si_002.pdf]

Synthesis and Evaluation of a Series of Ni(II) Complexed Nucleophilic Glycine Equivalents

Audrey Jergensen[a], Emily Burgess[a], Mackenzie Bergagnini[a], Delaney McDonald[a], Shawna B. Ellis[a], Trevor K. Ellis[a*]

[a]Department of Chemistry and Physics, Southwestern Oklahoma State University, 100 Campus Drive, Weatherford, OK 73096, USA

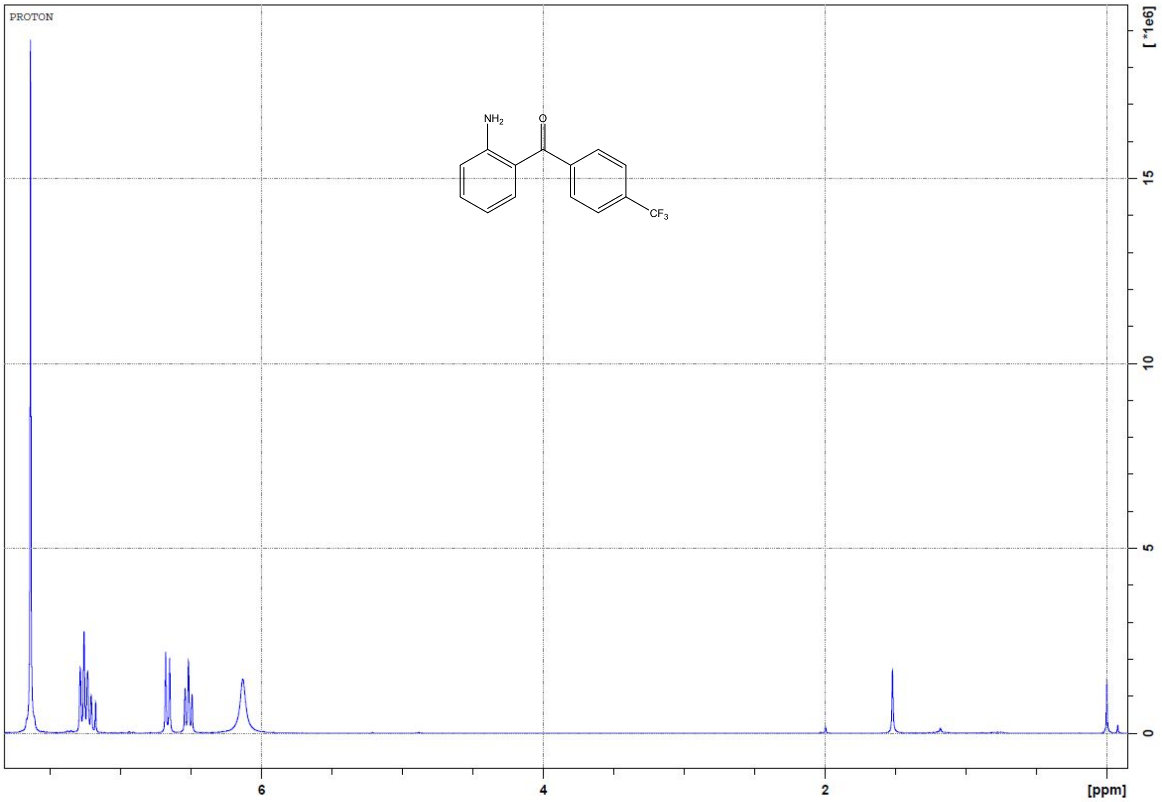

Figure S1: ^{1}HNMR spectra of **2-[4-(trifluoromethyl)benzoyl]aniline 1d**.

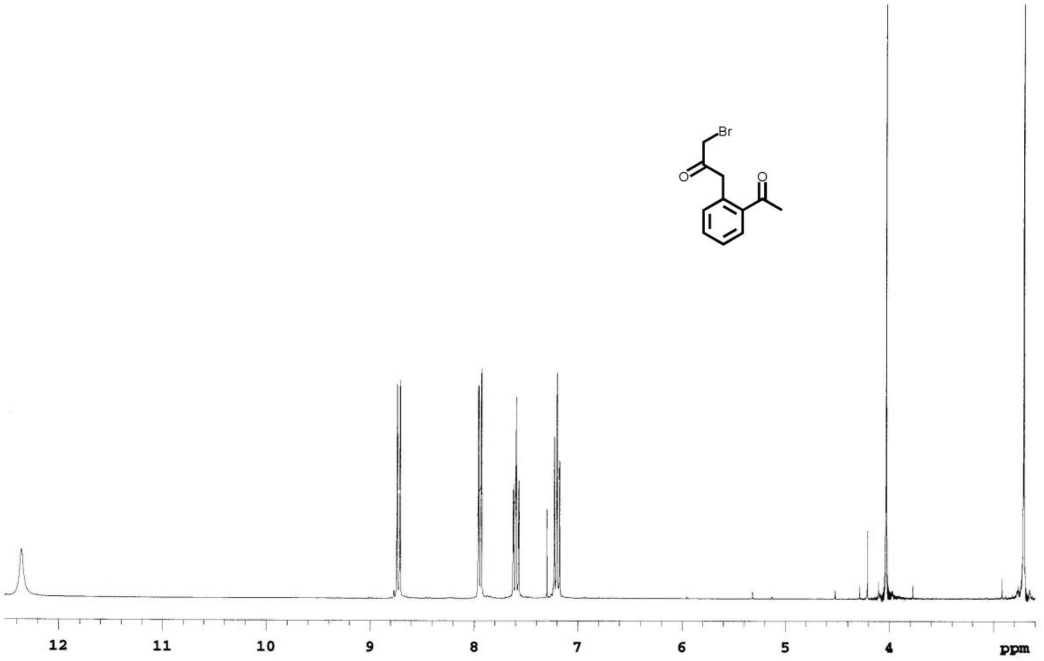

Figure S2: ^{1}HNMR spectra of ***N*-(2-acetylphenyl)-2-bromoacetamide 2a**.

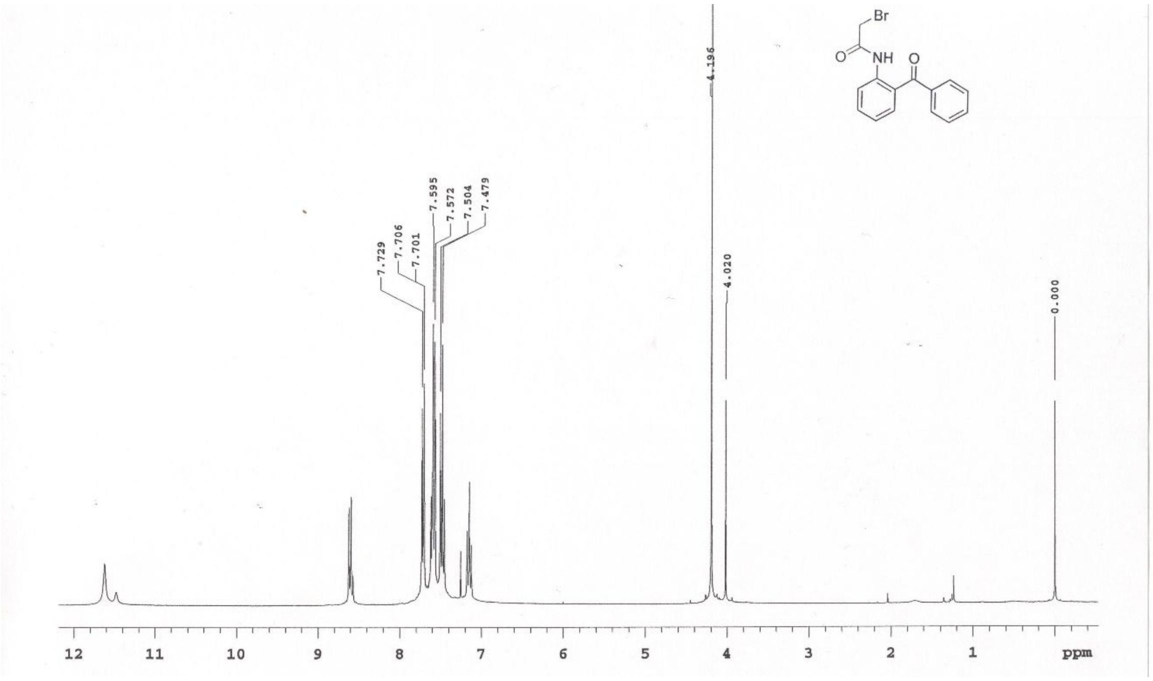

Figure S3: ^{1}HNMR spectra of ***N*-(2-benzoyl-phenyl)-2-bromo-acetamide 2b**.

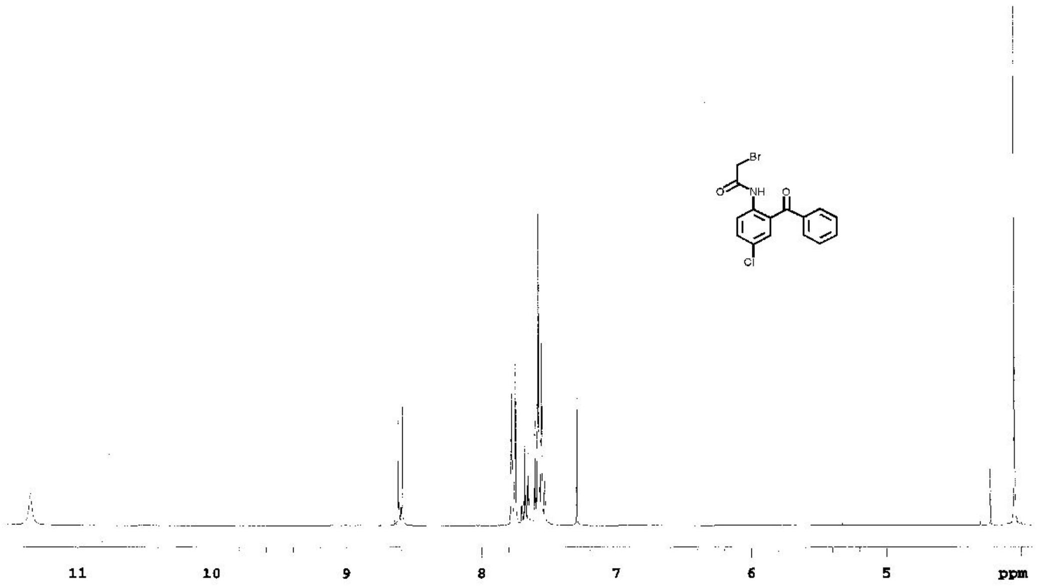

Figure S4: ^{1}HNMR spectra of ***N*-(2-benzyol-4-chlorophenyl)-2-bromoacetamide 2c**.

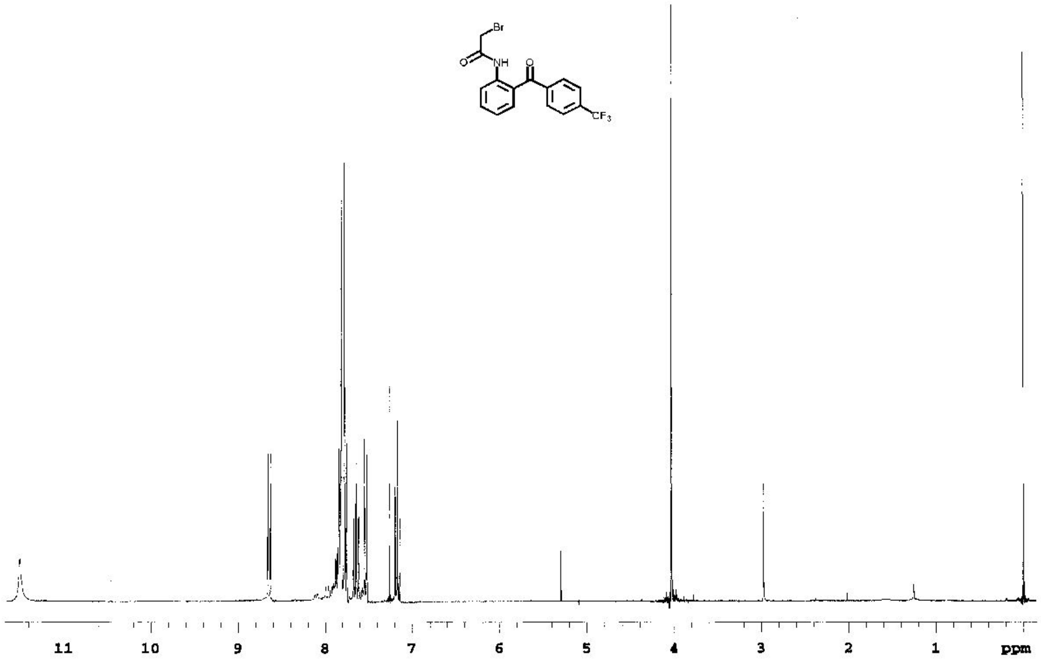

Figure S5: ^{1}HNMR spectra of ***N*-(2-(4-(1,1,1-trifluoromethyl)-benzoyl-phenyl)-2-bromoacetamide 2d**.

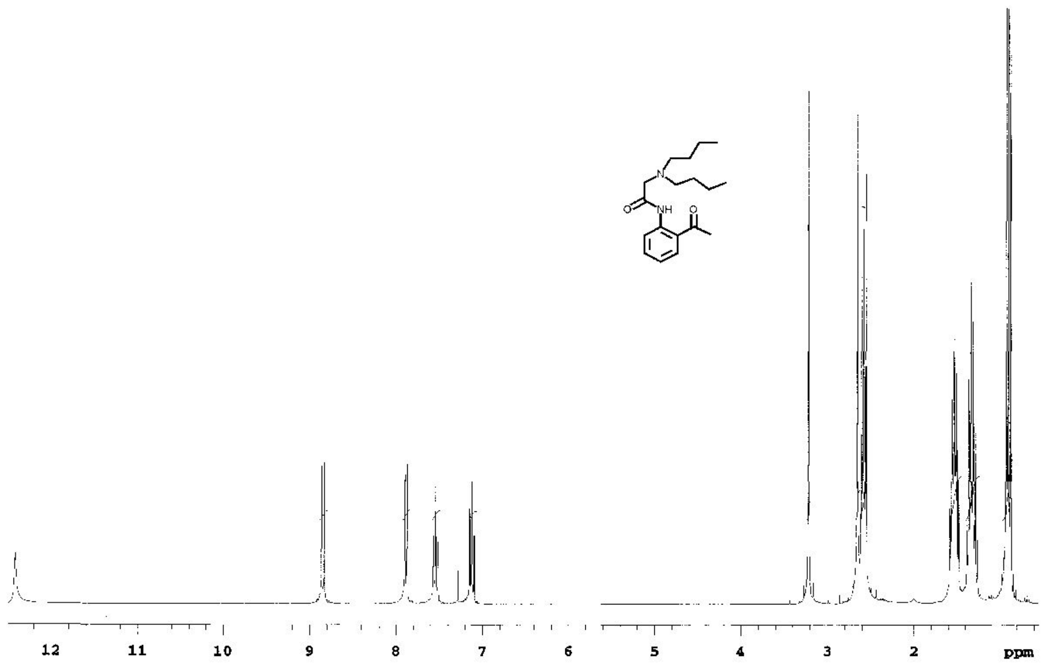

Figure S6: ^{1}HNMR spectra of ***N*-(2-acetyl-phenyl)-2-dibutylamino-acetamide 3a**.

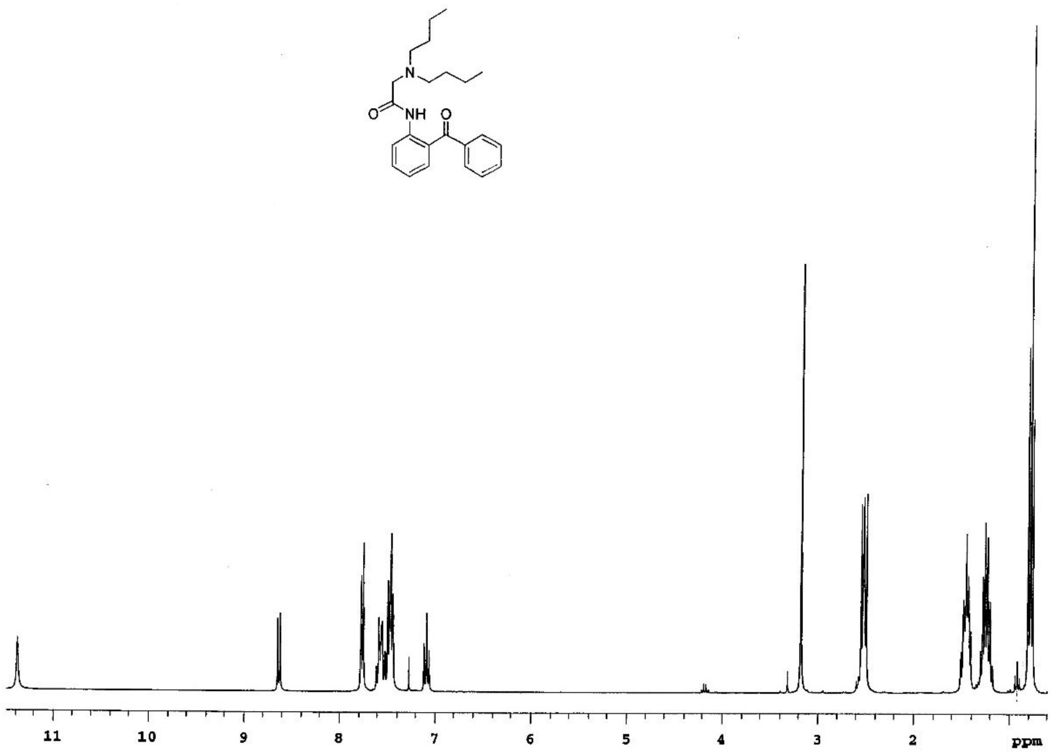

Figure S7: ^{1}HNMR spectra of ***N*-(2-benzoyl-phenyl)-2-dibutylamino-acetamide 3b**.

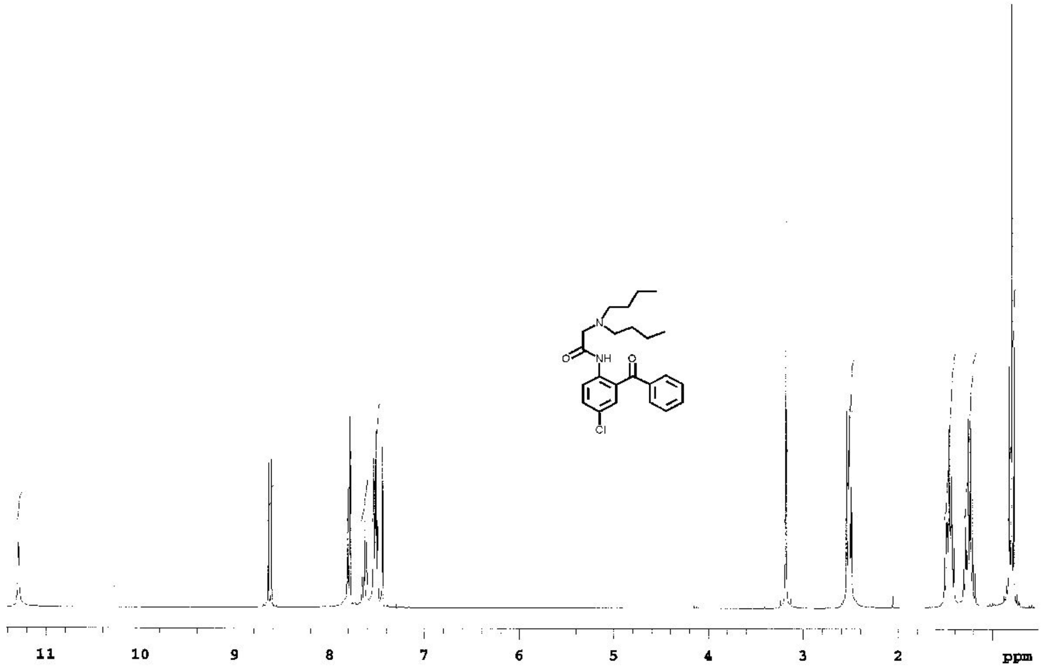

Figure S8: ^{1}HNMR spectra of ***N*-(2-benzoyl-4-chlorophenyl)-2-dibutylamino-acetamide 3c**.

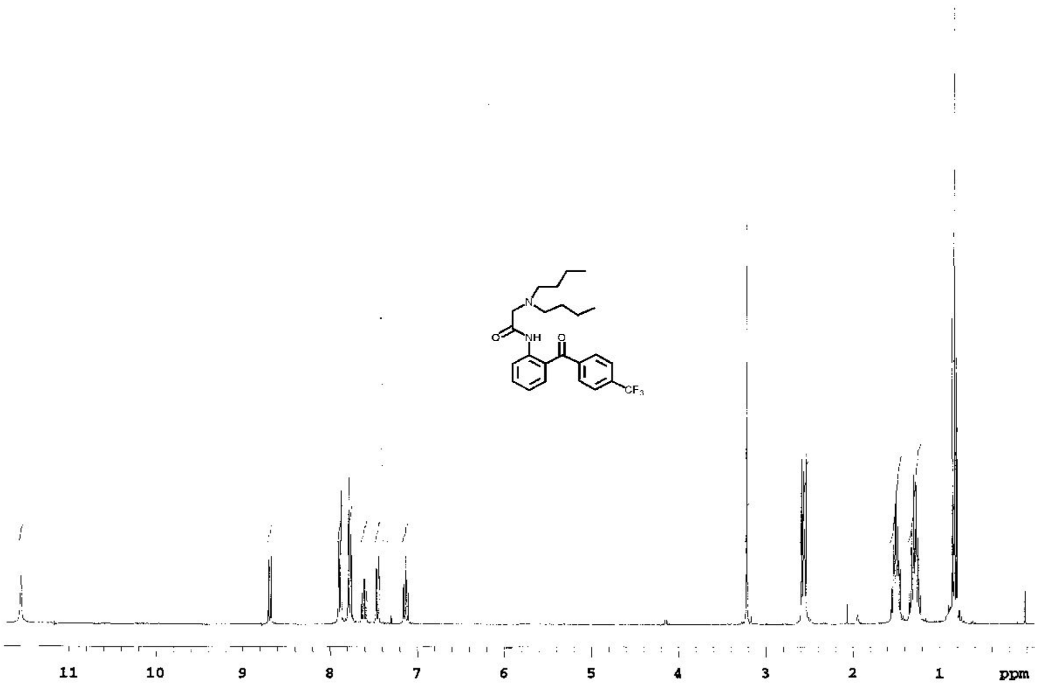

Figure S9: ^{1}HNMR spectra of ***N*-(2-(4-trifluoromethyl-benzoyl)-phenyl)-2-dibutylamino-acetamide 3d**.

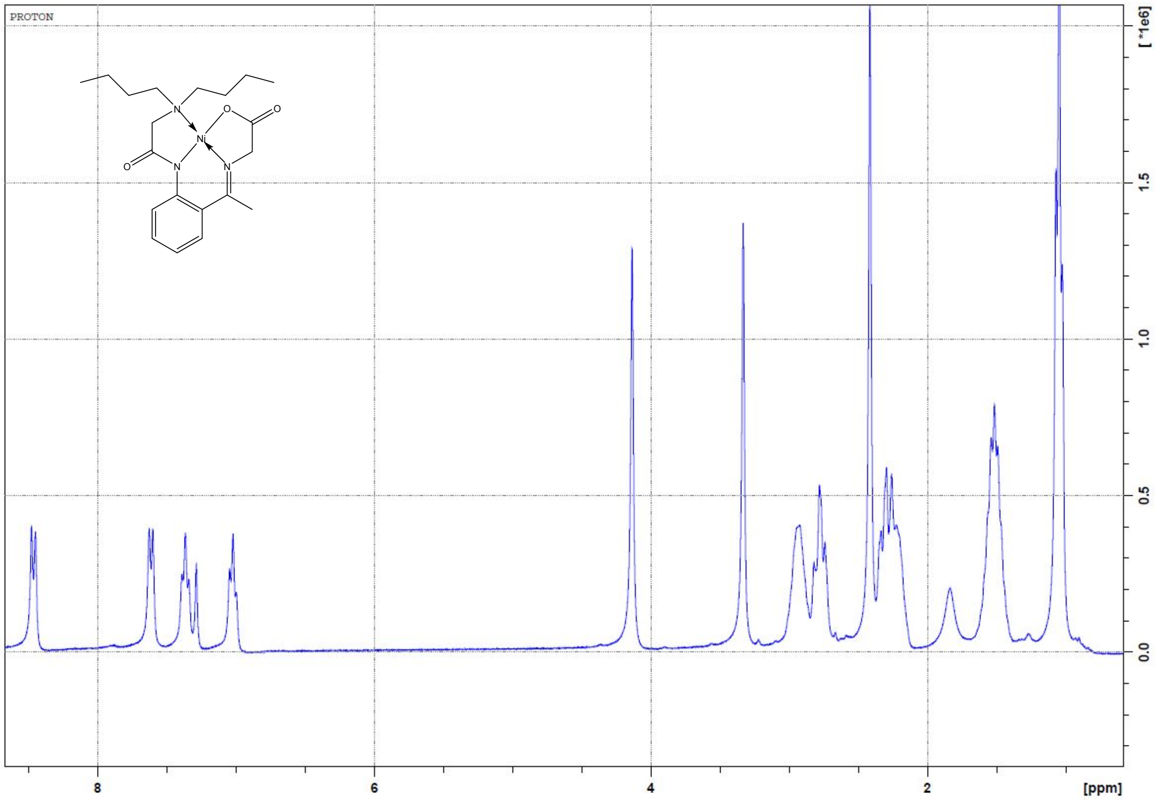

Figure S10: ^{1}HNMR spectra of **Ni(II) Complex of Glycine Schiff Base with *N*-(2-acetyl-phenyl)-2-dibutylamino-acetamide 4a**.

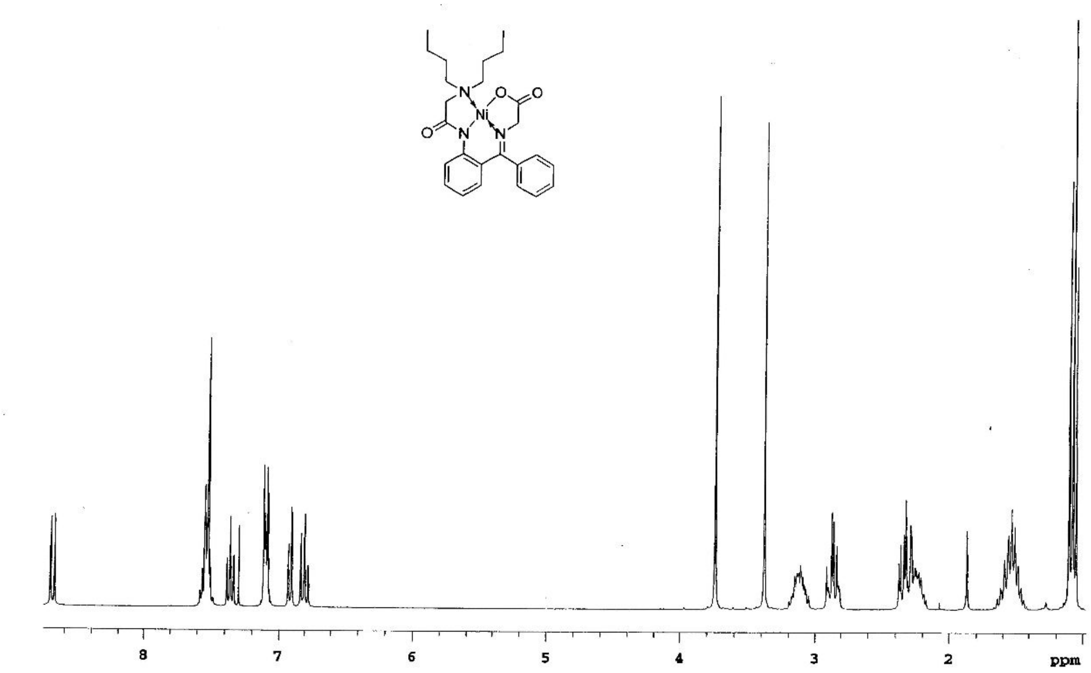

Figure S11: ^{1}HNMR spectra of **Ni(II) Complex of Glycine Schiff Base with *N*-(2-benzyoyl-phenyl)-2-dibutylamino-acetamide 4b**.

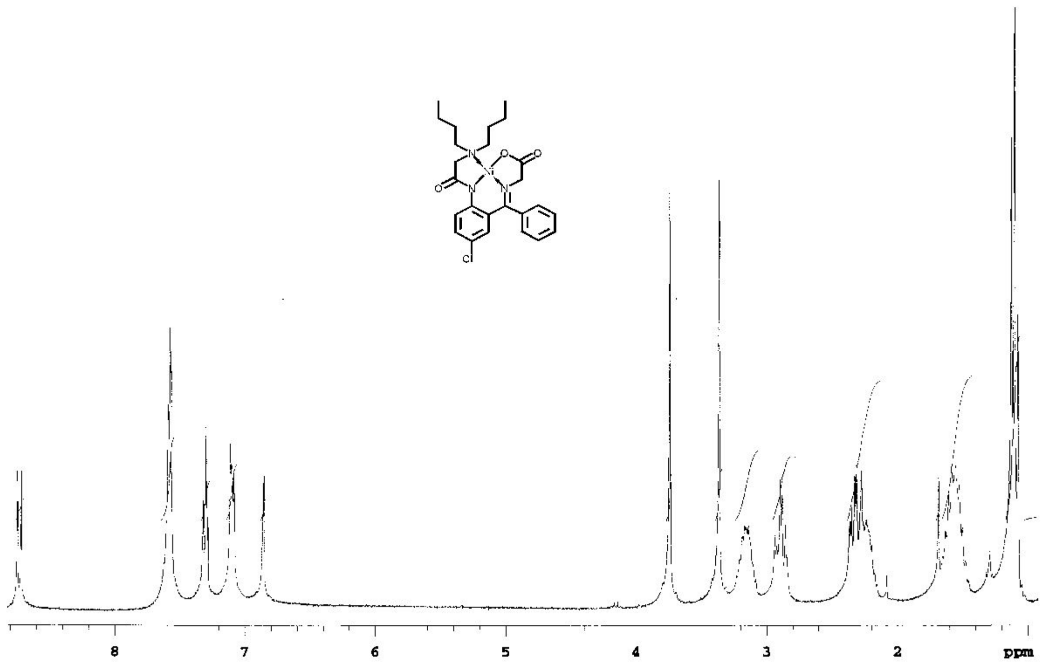

Figure S12: ^{1}HNMR spectra of **Ni(II) Complex of Glycine Schiff Base with *N*-(2-benzyoyl-4-chlorophenyl)-2-dibutylamino-acetamide 4c**.

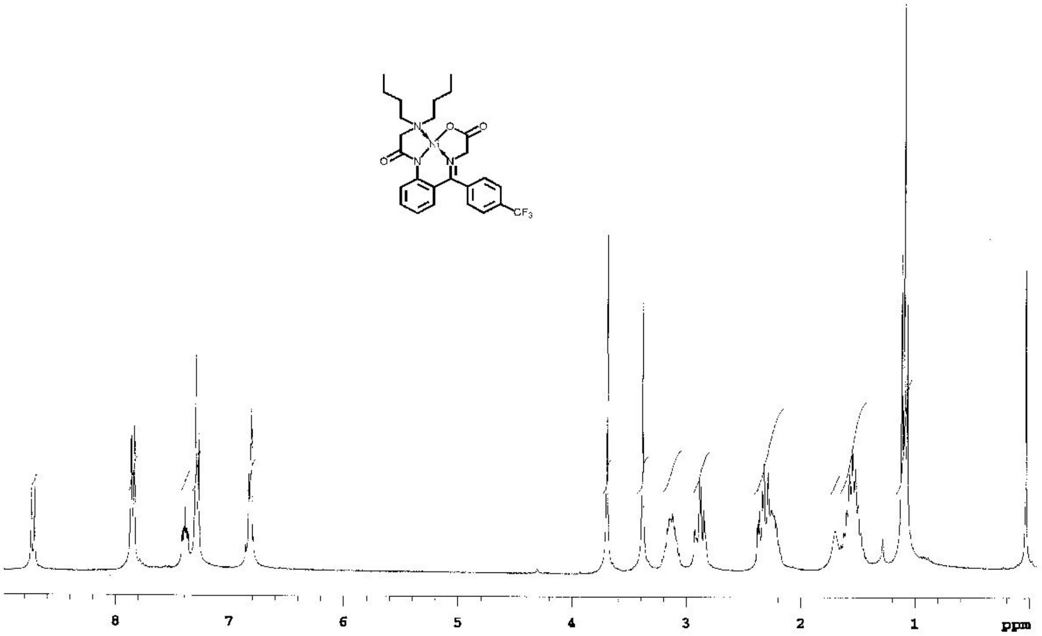

Figure S13: ^{1}HNMR spectra of **Ni(II) Complex of Glycine Schiff Base with *N*-(2-(4-1,1,1-trifluoromethyl)-benzoyl-phenyl)-2-dibutylamino-acetamide 4d**.

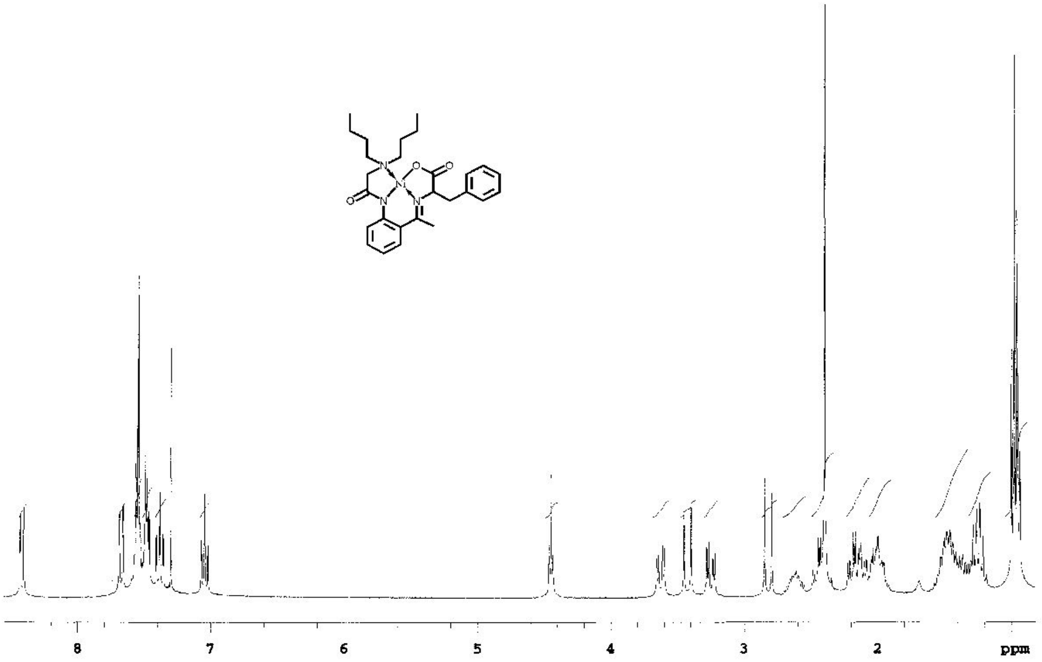

Figure S14: ^{1}HNMR spectra of **Ni(II) Complex of Phenylalanine Schiff Base with *N*-(2-acetyl-phenyl)-2-dibutylamino-acetamide 5a**.

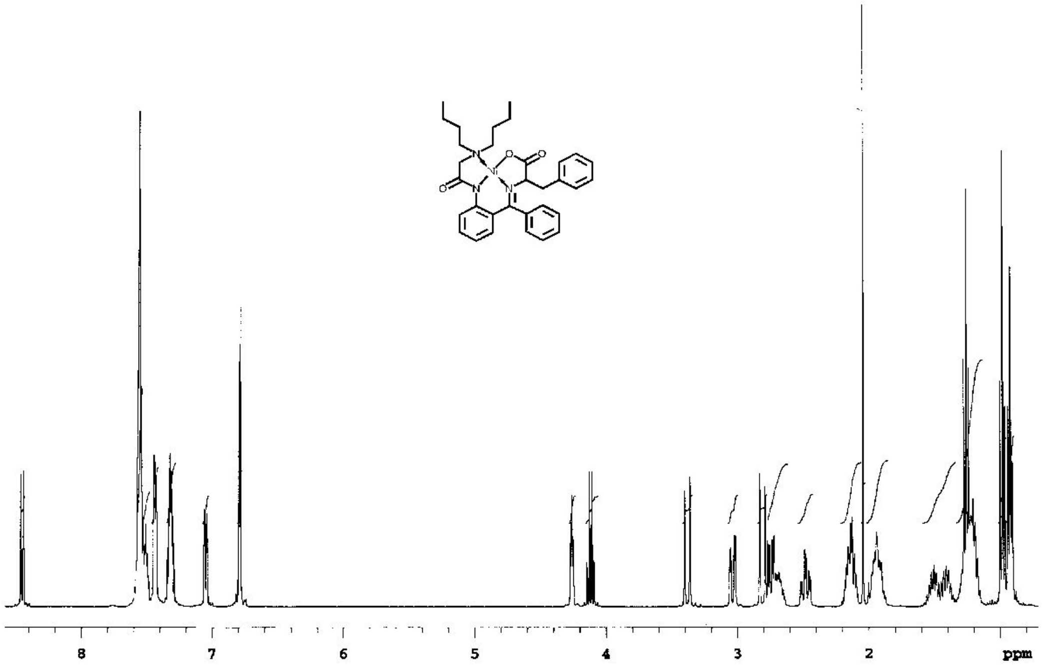

Figure S15: ^{1}HNMR spectra of **Ni(II) Complex of Phenylalanine Schiff Base with *N*-(2-benzoyl-phenyl)-2-dibutylamino-acetamide 5b**.

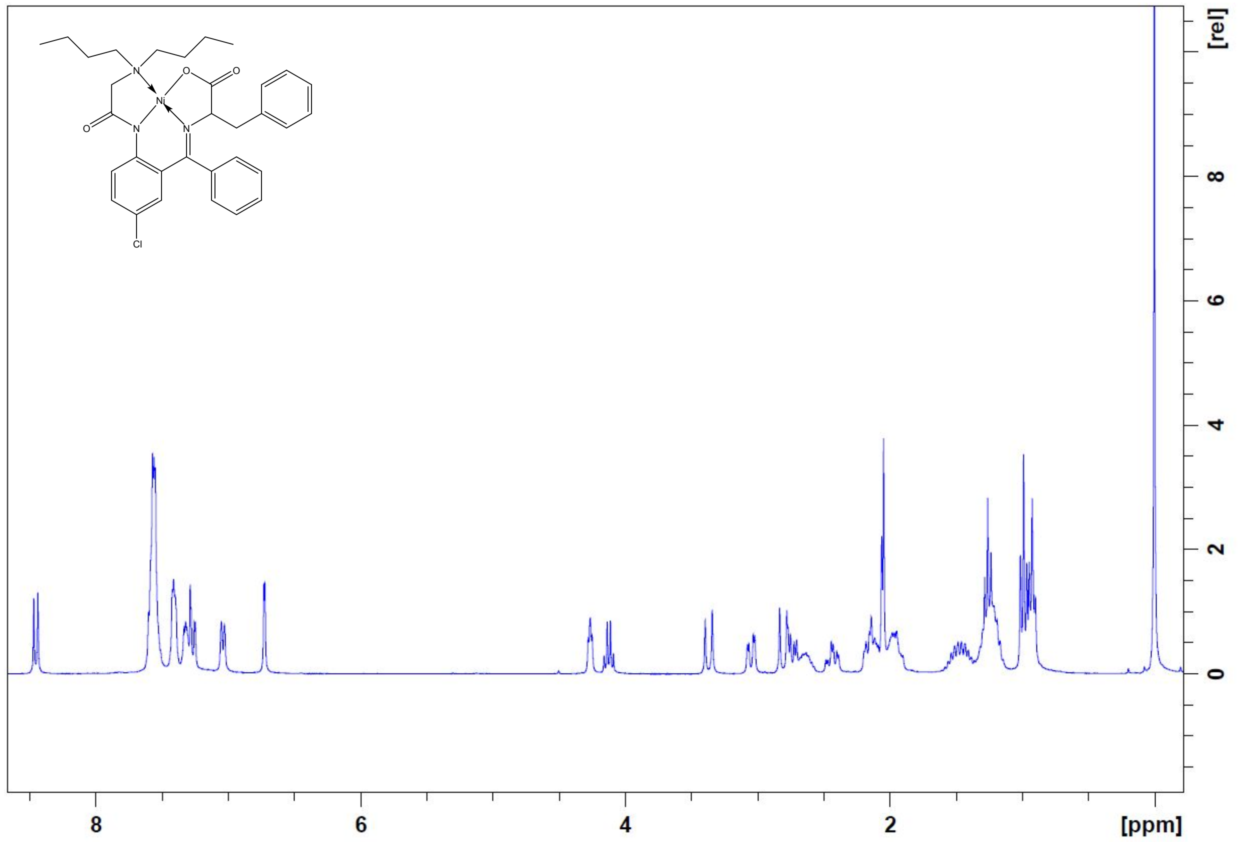

Figure S16: ^{1}HNMR spectra of **Ni(II) Complex of Phenylalanine Schiff Base with *N*-(2-benzoyl-4-chlorophenyl)-2-dibutylamino-acetamide 5c**.

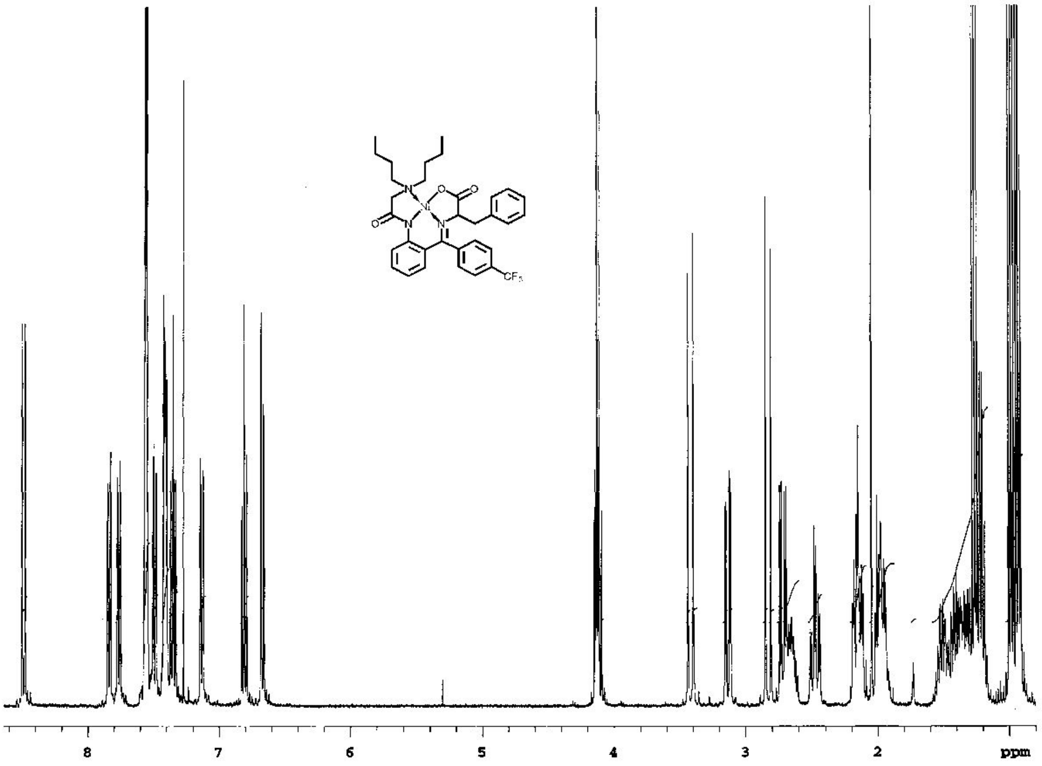

Figure S17: ¹HNMR spectra of **Ni(II) Complex of Phenylalanine Schiff Base with *N*-(2-(4-trifluoromethylbenzoyl)-phenyl)-2-dibutylamino-acetamide 5d**.

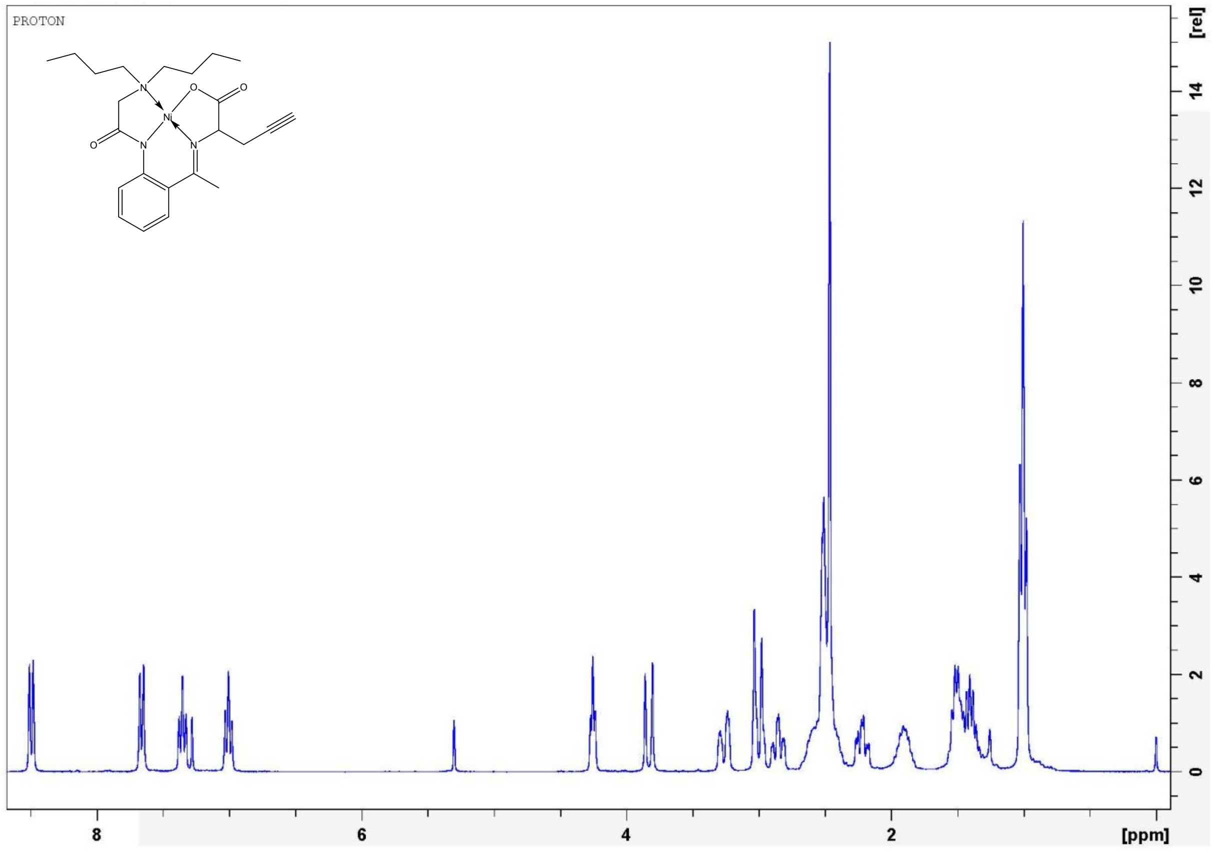

Figure S18: ^{1}HNMR spectra of **Ni(II) complex of 2-amino-pent-4-ynoic acid Schiff base with *N*-(2-acetyl-phenyl)-2-dibutylamino-acetamide 6a**.

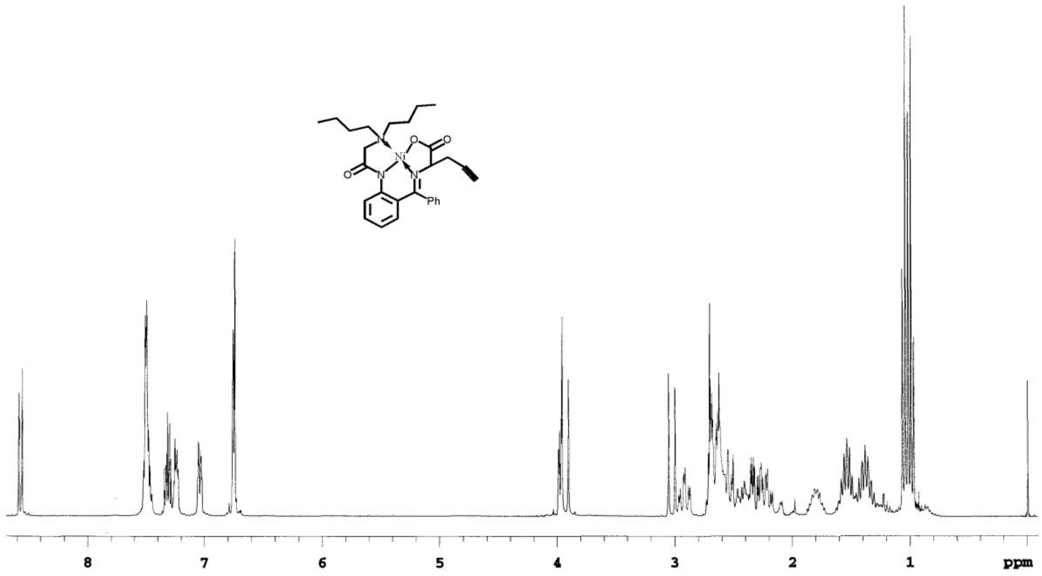

Figure S19: ^{1}HNMR spectra of **Ni(II) complex of 2-amino-pent-4-ynoic acid Schiff base with *N*-(2-benzoyl-phenyl)-2-dibutylamino-acetamide 6b**.

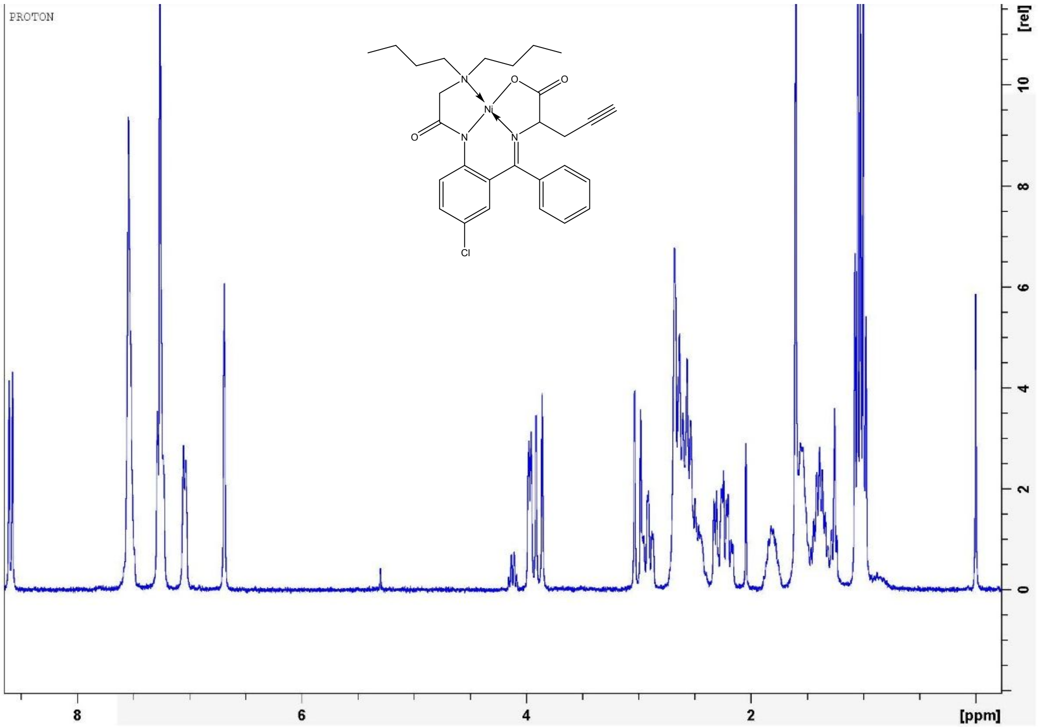

Figure S20: ^{1}HNMR spectra of **Ni(II) complex of 2-amino-pent-4-ynoic acid Schiff base with *N*-(2-benzoyl-phenyl)-2-dibutylamino-acetamide 6b**.

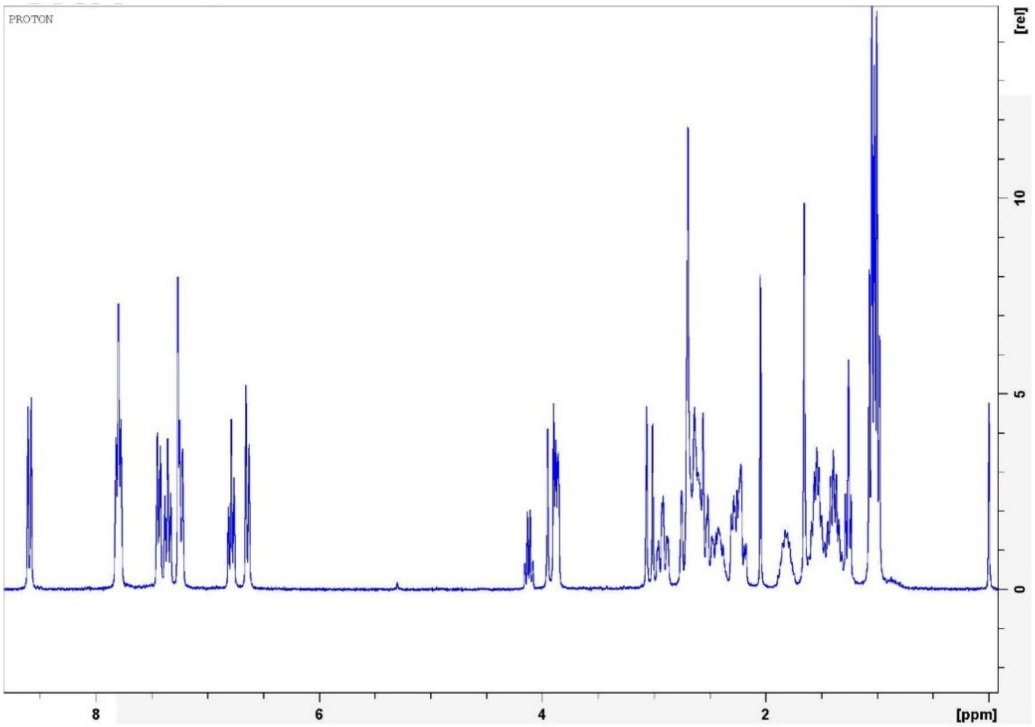

Figure S21: ^{1}HNMR spectra of **Ni(II) Complex of 2-amino-pent-4-ynoic acid Schiff Base with *N*-(2-benzoyl-4-chlorophenyl)-2-dibutylamino-acetamide 6c**.

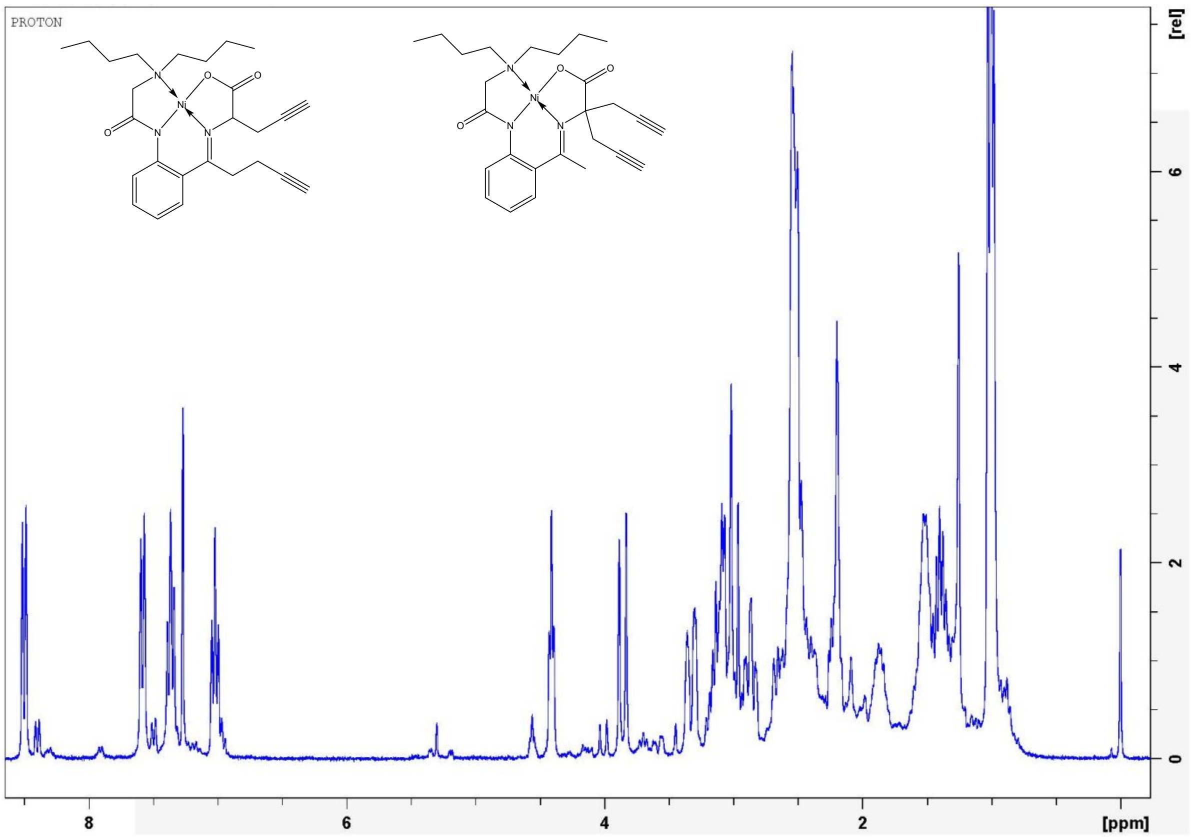

Figure S22: ^{1}HNMR spectra of mixture of **Ni(II) complex of 2-amino-pent-4-ynoic acid Schiff base with *N*-(2-(3-butynyl)-phenyl)-2-dibutylamino-acetamide and Ni(II) complex of 2-amino-2-prop-2-ynyl-pent-4-ynoic acid Schiff base with *N*-(2-acetyl-phenyl)-2-dibutylamino-acetamide 7a**.

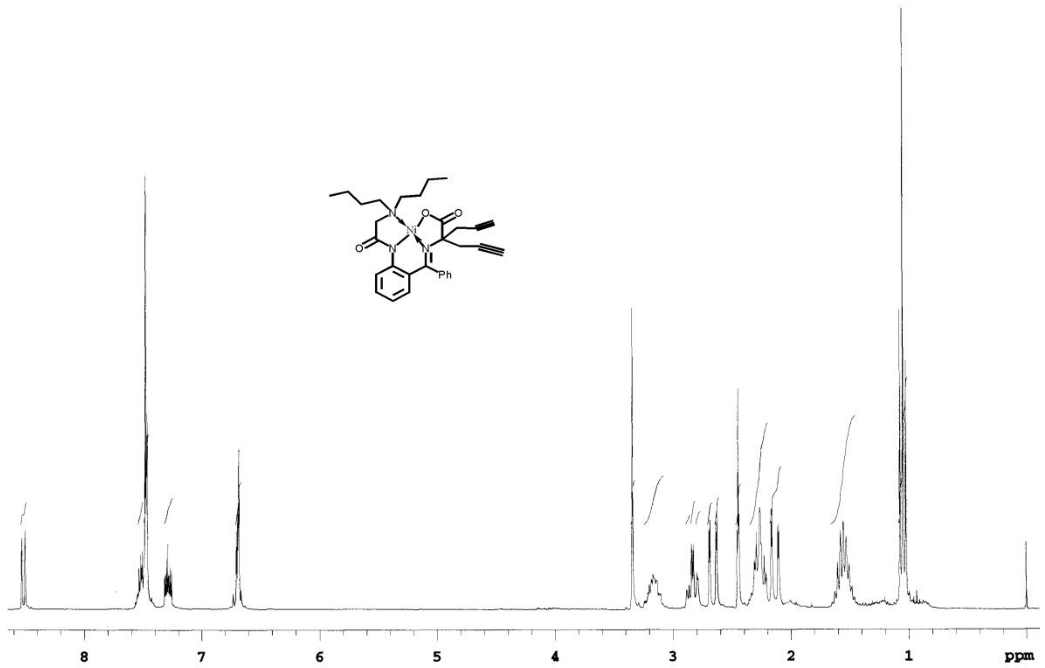

Figure S23: ^{1}HNMR spectra of **Ni(II) complex of 2-amino-2-prop-2-ynyl-pent-4-ynoic acid Schiff base with *N*-(2-benzoyl-phenyl)-2-dibutylamino-acetamide 7b**.

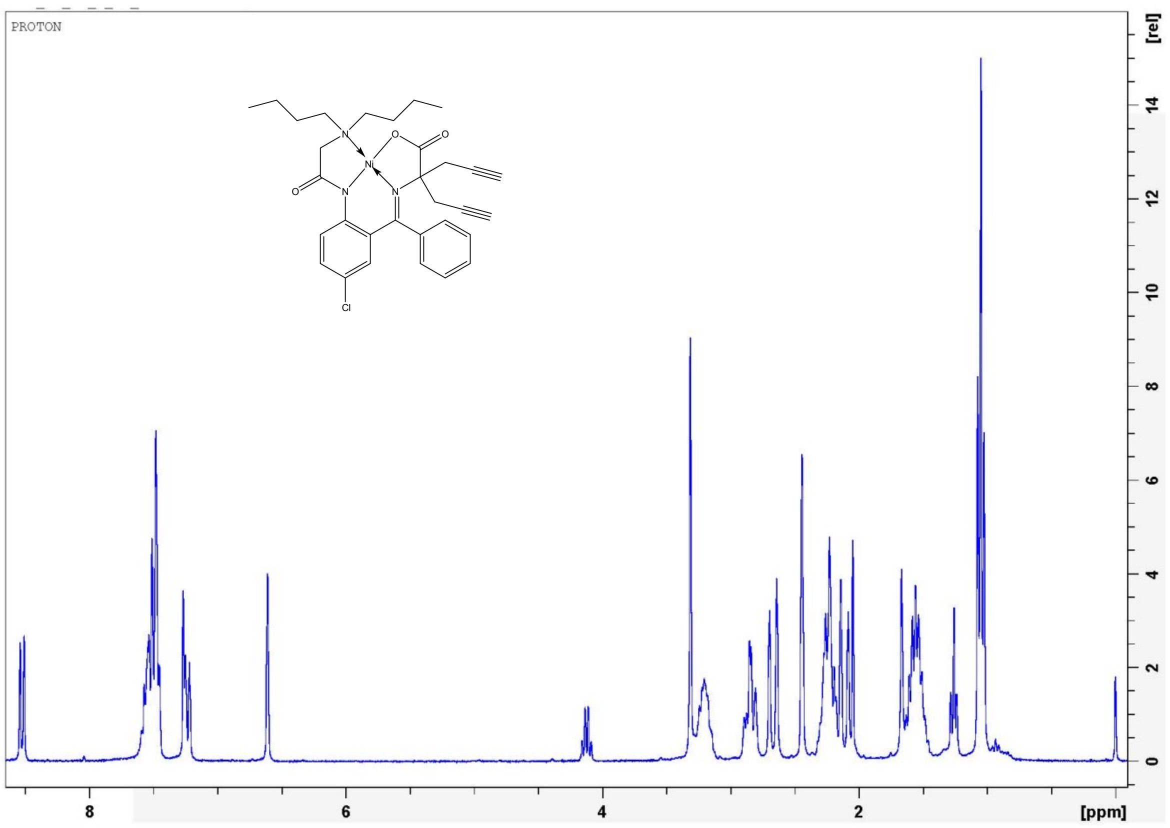

Figure S24: ^{1}HNMR spectra of **Ni(II) Ni(II) Complex of 2-amino-2-prop-2ynyl-pent-4-ynoic acid Schiff Base with *N*-(2-benzoyl-4-chlorophenyl)-2-dibutylamino-acetamide 7c**.

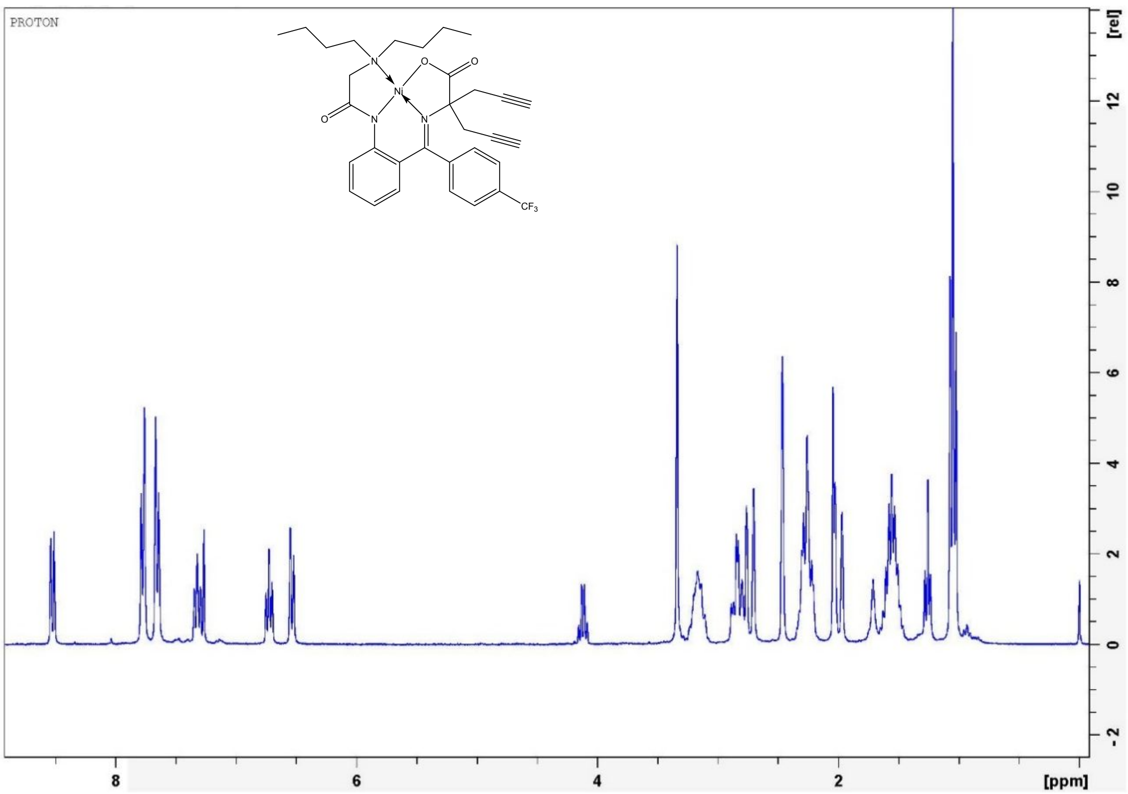

Figure S25: ^{1}HNMR spectra of **Ni(II) Complex of 2-amino-2-prop-2ynyl-pent-4-ynoic acid Schiff Base with *N*-(2-(4-trifluoromethylbenzoyl)-phenyl)-2-dibutylamino-acetamide 7d**.
